# Chimeric PRR T-cell-engager targeting cell surface β-1,3-glucan for invasive candidiasis

**DOI:** 10.1371/journal.ppat.1013508

**Published:** 2025-09-15

**Authors:** Yu Fang Sun, Shi Yu Guo, Si Qi Wang, Rui Tong Li, Xi Ran Qiu, Xing Chen Dong, Shuang Liu, Hui Shen, Mao Mao An

**Affiliations:** 1 Department of Pharmacology, Shanghai Tenth People’s Hospital, Tongji University School of Medicine, Shanghai, China; 2 Starmab Biologics (Suzhou) Co., Ltd., Shanghai, China; 3 Department of Clinical Laboratory Medicine, Shanghai Tenth People’s Hospital, Tongji University School of Medicine, Shanghai, China; Rutgers New Jersey Medical School, UNITED STATES OF AMERICA

## Abstract

Invasive candidiasis, primarily caused by *Candida albicans*, represents the most common fungal disease among hospitalized patients and poses a significant threat to human health. Intrinsic or acquired immunosuppression serves as a critical risk factor predisposing individuals to this disease, while simultaneously reducing the efficacy of conventional antifungal therapies and worsening clinical outcomes. Given the central role of immune dysfunction in the pathogenesis of invasive candidiasis, immunotherapeutic strategies hold substantial promise. We targeted dectin-1, the primary pattern recognition receptor for β-1,3-glucan, by engineering XJ104, a bispecific T-cell engager that fuses dectin-1 to the light chains of an anti-CD3 monoclonal antibody. This construct is designed to bridge *Candida* β-1,3-glucan with CD3 on T cells, thereby inducing anti-*Candida* immunity. Our results demonstrate that XJ104 exhibits high specificity for β-1,3-glucan and activates effector cells in a *Candida*-dependent manner *in vitro*. In murine models, XJ104 enhances Th1 and Th17 responses and confers significant protection against both *C. albicans* and *non-albicans* infections. Crucially, CD3^+^ T-cell depletion and cytokine neutralization abolished this protection, confirming the T-cell-dependent protective efficacy of XJ104. These results establish that enhancing the endogenous T-cell function represents an effective strategy against invasive candidiasis. In conclusion, our study presents a novel therapeutic approach that bridges T cells and *Candida* pathogens, promoting robust *Candida*-specific immunity and controlling invasive infections caused by *Candida spp*. These findings underscore the potential of XJ104 as a clinically promising immunotherapy for the treatment of invasive candidiasis.

## Author summary

Invasive candidiasis represents a life-threatening challenge for immunocompromised patients, necessitating therapies that enhance antifungal immunity. Here, we developed XJ104, a T cell engager that bridges between *Candida* β-1,3-glucan and CD3 on T cells to redirect endogenous T-cell activity against *Candida* pathogens. Mechanistically, XJ104 mediates protection through T-cell activation and Th1/Th17 polarization, augmenting production of IFN-γ and IL-17, cytokines essential for fungal clearance, as validated in murine candidemia models. Our findings establish proof-of-concept for a dectin-1-based T cell engager as a novel immunotherapeutic strategy that leverages endogenous T cells to combat invasive candidiasis. This approach holds translational promise for addressing the rising threat of invasive fungal infections in clinical settings.

## Introduction

Invasive candidiasis, primarily caused by *Candida albicans*, represents the most common fungal disease among hospitalized patients, encompassing a spectrum of disorders that predominantly affect immunocompromised or critically ill individuals [1,2]. *Candida* species routinely colonize healthy hosts without causing disease, maintained by a precise host immunity-fungal pathogen balance [1,3,4]. Disruption of this homeostasis, frequently due to immune dysfunction, creates conditions favoring *Candida* overgrowth, enabling hematogenous dissemination and subsequent invasion of organs, particularly the kidneys, liver, and heart [5,6]. Immune impairment not only constitutes a major risk factor for disease development but also contributes to the failure of conventional antifungal therapies. Despite antifungal treatment, the mortality rate remains approximately 40% in patients with invasive candidiasis [7–9]. The emergence of multidrug-resistant strains, such as *C. auris* and *C. parapsilosis,* further complicates clinical management due to their reduced susceptibility to standard antifungal agents [1]. Recognizing this growing threat, the World Health Organization (WHO) issued its first list of 19 fungal priority pathogens on October 25, 2022, classifying *C. albicans* and *C. auris* as the critical priority group. This initiative underscores the urgent need for intensified global efforts against these escalating public health challenges [10].

Invasive candidiasis typically arises from aberrant fungal colonization coupled with compromised host defense mechanisms, either localized or systemic [11]. Notably, unlike mucocutaneous candidiasis, many patients affected by invasive candidiasis do not have a known immunodeficiency, such as IL-17 and Th17 signaling pathways [1,12]. Nevertheless, mucocutaneous candidiasis can also progress to more severe and invasive forms in susceptible individuals [1], such as prolonged or recurrent broad-spectrum antibiotic use [5,13], iatrogenic immunosuppression [5], and compromised integrity of gastrointestinal and cutaneous barriers [11]. Host protection against *Candida* infection is mediated through both Th1 and Th17 immune response. While individuals with impaired IL-17 signaling exhibit heightened susceptibility to mucosal candidiasis but not to invasive disease, IL-17 plays a critical role in neutrophil recruitment and activation, which is essential for clearance of invasive *Candida* [14]. Supporting this, a prospective case series demonstrated that adjunctive recombinant IFN-γ therapy promoted recovery from invasive fungal infections in candidemia patients, correlating with enhanced IL-17 and IL-22 production [15]. One study revealed that IL-17 regulates systemic fungal immunity by functionally modulating natural killer (NK) cells [16]. Mounting evidence confirms the indispensable role of IL-17 in controlling systemic candidiasis [16–18]. Collectively, these findings highlight the promise of T-cell-based immunotherapy for invasive candidiasis management [14,19,20].

T-cell-based immunotherapies, particularly T-cell engagers (TCEs) that simultaneously target the CD3 subunit of the T-cell receptor (TCR) and tumor-associated antigens (TAAs), have revolutionized cancer treatments [21]. By engaging both targets, these bispecific molecules activate CD3 signaling, inducing immune synapse formation and the secretion of cytolytic proteins [22–24]. This platform may also prove effective against infectious diseases [25–27]. Given the established mechanism of action (MOA) and clinical success of TCEs in oncology, we hypothesize that TCEs hold therapeutic potential for controlling invasive candidiasis.

β-1,3-glucan, a key polysaccharide component of the fungal cell wall, serves as a pathogen-associated molecular pattern (PAMP) recognized by dectin-1, a critical pattern recognition receptor (PRR) expressed on host innate immune cells [28–31]. Here, we developed XJ104, a chimeric PRR-based TCE that fuses the P119-M247 carbohydrate-binding domain of human dectin-1 with an anti-CD3 monoclonal antibody. We demonstrate that XJ104 bridges T cells and *Candida* pathogens by simultaneously engaging CD3 and β-1,3-glucan, thereby inducing *Candida*-specific, T-cell mediated immune responses *in vitro* and *in vivo*. Notably, XJ104 conferred protection against candidemia in murine models. These findings establish XJ104 as a novel and promising therapeutic candidate for invasive candidiasis in immunocompromised patients.

## Results

### The 119–247aa domain of dectin-1 is optimal for T cell engager construction

**Fig 1:**
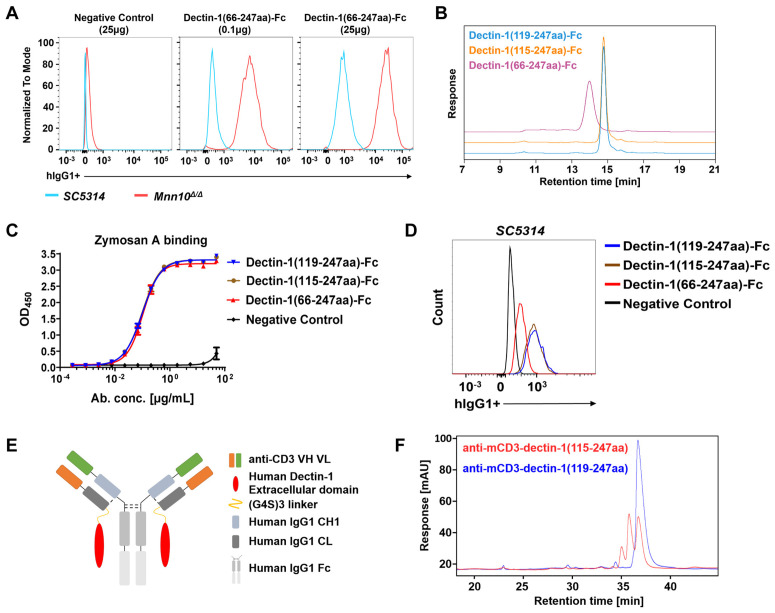
The extracellular domain 119-247aa of dectin-1 represents the optimal region for engineering T cell engager. (A) Binding of dectin-1(66-247aa)-Fc to both *C. albicans SC5314* and *Mnn10*^*Δ/Δ*^, as determined by flow cytometry. (B) The overlay SEC profiles of dectin-1-Fc fusion proteins. (C-D) Binding of dectin-1-Fc fusion proteins to zymosan A, as evidenced by ELISA (C) and flow cytometry (D). (E) Structural diagram of T cell engager. (F) The purity of T cell engager was assessed by NR-CE. Data are representative of three independent experiments (A, B, D, F). Data are means±SD (n = 2) and are representative of three independent experiments (C). SEC, size exclusion chromatography; ELISA, enzyme-linked immunosorbent assay; NR-CE, non-reducing capillary electrophoresis.

Dectin-1 is a type II transmembrane protein with glycosylation [32]. Because inappropriate glycosylation of therapeutic antibodies can lead to aggregation and degradation, compromising pharmacokinetics, efficacy, and safety [33], we first sought to identify the optimal dectin-1 domain for TCE construction. We generated a dectin-1(66–247aa)-Fc fusion protein by fusing the extracellular domain of dectin-1 (amino acids 66–247) to human IgG1 Fc (S1A Fig) [34]. Using the *C. albicans Mnn10* mutant (*Mnn10*^*Δ/Δ*^), which exhibits more exposed surface β-1,3-glucan than the *C. albicans* parental strain [35], we confirmed that dectin-1(66–247aa)-Fc showed higher affinity for *Mnn10*^*Δ/Δ*^ (Fig 1A). However, mass spectrometry failed to detect fragment ions spanning amino acids 90–111 in the stalk region (66–114aa), and the intact molecular weight of dectin-1(66–247aa)-Fc (S1B, S1C, S1D and S1E Fig) [34]. While the fusion protein bound *C. albicans*, the inability to verify its intact structure raises concerns about its structural integrity.

Trypsin digestion of the dectin-1(66–247aa) domain yielded 12 expected peptide fragments. Mass spectrometry confirmed 11 fragments matched their predicted sequences, with only the 90–111aa fragment undetected (S1C Fig). Representative spectra for adjacent fragments are shown in S1B and S1D Fig. Based on these results, we deleted the stalk region (containing the 90–111aa) and constructed dectin-1(115–247aa)-Fc, retaining only the carbohydrate-recognition domain (CRD, 115–247aa) [34]. Mass analysis of dectin-1(115–247aa)-Fc correctly detected a molecular weight of 84,329 Da, but revealed unexpected cleavage of *N*-terminal amino acids (115V/116L/117S), resulting in an 84,031 Da product (S1F Fig). To address this, we generated dectin-1(119–247aa)-Fc, eliminating the cleavage site (115V/116L/117S-118S). This construct showed a molecular weight of 83,618 Da, matching its theoretical sequence and confirming proper primary structure, though partial loss of the N-terminal proline (position 119) was observed (S1G Fig).

Protein purity and binding capacity were paramount considerations for recombinant dectin-1 constructs. Size exclusion chromatography (SEC) demonstrated typical monomeric profiles for both dectin-1(119–247aa)-Fc and dectin-1(115–247aa)-Fc, whereas dectin-1(66–247aa)-Fc exhibited significant peak broadening (Fig 1B). Consistent with this, reducing polyacrylamide gel electrophoresis confirmed the expected molecular weights (~42 kDa) for dectin-1(119–247aa)-Fc and dectin-1(115–247aa)-Fc, while dectin-1(66–247aa)-Fc showed aberrant migration (~47 kDa vs. 55 ~ 70 kDa) (S1H Fig). Based on prior research, we hypothesized that the post-translational modifications in the stalk region might cause protein aggregation or degradation, explaining both the reduced purity and previously reported mass spectrometry detection failures.

To evaluate the binding capacity of dectin-1-Fc fusion proteins, we used zymosan A as a soluble antigen and *C. albicans SC5314* as a cellular target. Enzyme-linked immunosorbent assay (ELISA) revealed an EC_50_ of 100.7 ng/mL for dectin-1(119–247aa)-Fc binding to zymosan A, with no significant affinity differences observed for the other two constructs (Fig 1C). Flow cytometry analysis showed that dectin-1(66–247aa)-Fc had slightly reduced binding to *C. albicans SC5314* compared to both dectin-1(119–247aa)-Fc and dectin-1(115–247aa)-Fc (Fig 1D). The CRD of dectin-1, highly conserved across variants, mediates β-1,3-glucan binding. Notably, the stalk region is absent in the short-splicing variant of dectin-1B [29,34]. These results demonstrate that neither stalk region deletion nor N-terminal CRD truncation significantly affects dectin-1’s β-1,3-glucan binding activity.

We next constructed chimeric PRR TCEs incorporating either the 115–247aa or 119–247aa of dectin-1. Using a developed monoclonal antibody against mouse CD3 (mCD3) that showed high affinity for mCD3 protein and specific binding to CD3-expressing murine splenocytes (S2 Fig), we engineered two TCEs by conjugating this anti-mCD3 antibody to truncated dectin-1 fragments (Fig 1E). Non-reduced capillary electrophoresis (NR-CE) analysis revealed that anti-mCD3-dectin-1(115–247aa) exhibited three distinct peaks while anti-mCD3-dectin-1(119–247aa) showed a single peak (Fig 1F). Collectively, these findings strongly indicate that the 119–247aa domain represents the optimal dectin-1 fragment for TCE construction compared to either the 66–247aa or 115–247aa domains.

### mXJ104 incorporating dectin-1(119–247aa) exhibits specific binding activity and induces polyfunctional effector T cells

The anti-mCD3-dectin-1(119–247aa) construct containing the Fc-region double mutation (L234A/L235A) was designated as mXJ104 and selected for further characterization. This is consistent with a previous study demonstrating that the L234A/L235A mutation abrogates binding to both FcγRs and C1q, thereby eliminating antibody-dependent cellular cytotoxicity (ADCC) and complement-dependent cytotoxicity (CDC) functions [36]. Our binding assays confirmed that mXJ104 exhibits no detectable interaction with FcγRI, FcγRIIa, FcγRIIb, or FcγRIIIa (S3 Fig). This result verifies the complete removal of effector functions.

**Fig 2:**
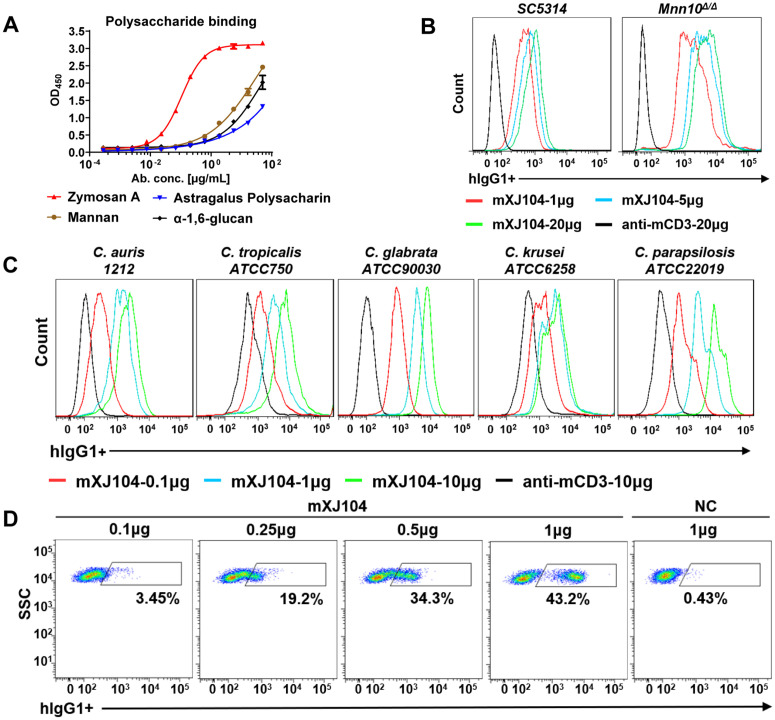
mXJ104 incorporating 119–247aa of dectin-1 binds to both *Candida* species and T cells. (A) ELISA assay of mXJ104 binding to zymosan A. Data are means ± SD (n = 2) and are representative of three independent experiments. (B, C, D) Cell-binding of mXJ104 to *C. albicans* (B), non-*albicans Candida* (C) and murine splenocytes (D). The fluorescence intensity was detected by flow cytometry. Data are representative of three independent experiments (B-D). ELISA, enzyme-linked immunosorbent assay.

Mass analysis determined the molecular weight of mXJ104 to be 176428 Da, matching the theoretical value calculated from its amino acid sequence (S4A Fig). Non-reducing polyacrylamide gel electrophoresis revealed a single band at ~180 kDa corresponding to the intact molecule, while under reducing conditions we observed two distinct bands: anti-mCD3 heavy chain (~55 kDa) and the light chain fused to dectin-1(119–247aa) (~40 kDa) (S4B Fig). ELISA demonstrated that mXJ104 specifically binds to zymosan A, which is rich in β-1,3-glucan, but not to other polysaccharides (Fig 2A). This specificity is particularly relevant given that β-1,3-glucan constitutes the primary structural component of cell walls in most fungi [29]. Indeed, mXJ104 showed comparable binding activity against multiple clinically relevant *Candida* species (Fig 2B and 2C), suggesting potential broad-spectrum antifungal activity. Importantly, mXJ104 maintained its expected binding capacity to CD3 subunits expressed on murine splenocytes (Fig 2D), confirming preservation of its anti-mCD3 functionality.

**Fig 3:**
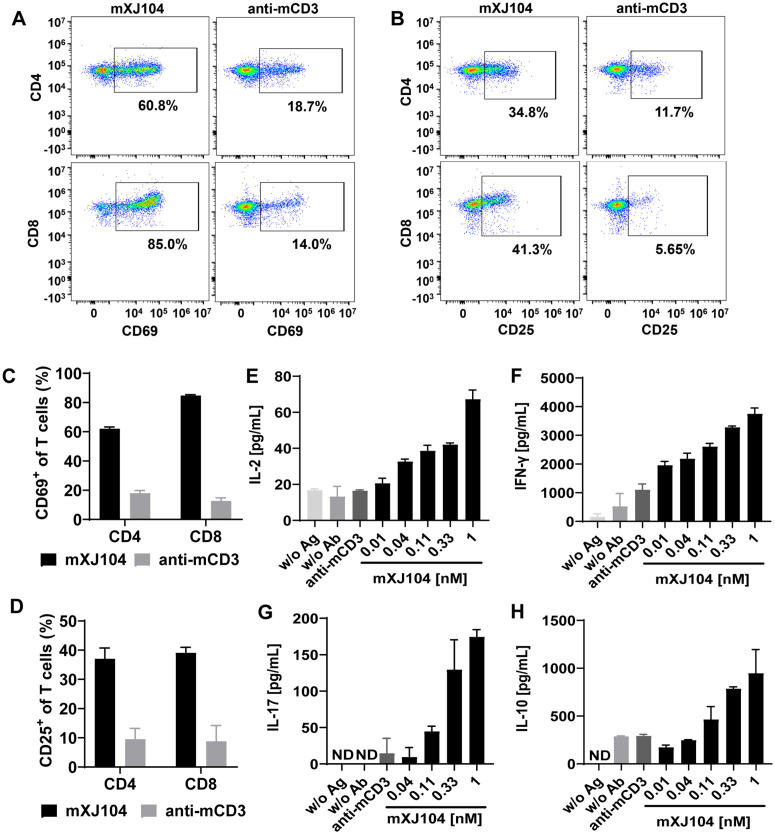
mXJ104 induces polyfunctional effector T cells. Murine splenocytes isolated from *C. albicans SC5314* infected-mice were culture on zymosan A-coated plates with mXJ104, anti-mCD3, w/o Ag or w/o Ab. (A-B) Exemplary flow cytometry analysis of CD69 (A) and CD25 (B) expression on CD4^+^ (top panels) and CD8^+^ (bottom panels) T cells after 20 hours. Example plots are gated on CD4^+^ (top panels) and CD8^+^ (bottom panels) T lymphocytes and are representative of three independent experiments. (C-D) The frequency of CD69^+^ (C) and CD25^+^ (D) T cells. Data are means ± SD (n = 3). (E-H) Cytokine concentrations measured by ELISA at indicated time points, IL-2 (E, 48 hours), IFN-γ (F, 48 hours), IL-17 (G, 168 hours), and IL-10 (H 48 hours) following treatment with increasing concentrations of mXJ104 or anti-mCD3, or controls. Data are means ± SD (n = 2) and are representative of three independent experiments (E-H). IFN-γ, interferon-γ; IL-, interleukin-; w/o Ag, without zymosan A but with mXJ104 (1 nM); w/o Ab, without mXJ104 and anti-mCD3, ND, no detection; ELISA, enzyme-linked immunosorbent assay.

To evaluate the T cell costimulatory capacity of mXJ104, freshly isolated murine splenocytes were cultured in zymosan A-coated plates with mXJ104 or anti-mCD3. Treatment with mXJ104 significantly upregulated T-cell activation markers CD69 and CD25 (Fig 3A, 3B, 3C and 3D), demonstrating effective T-cell activation. mXJ104 stimulation induced dose-dependent polarization of activated T cells along with increased production of IL-2, IFN-γ, IL-17, and IL-10, whereas anti-mCD3 treatment alone failed to elicit comparable cytokine responses. Of note, neither zymosan A nor mXJ104 monotherapy induced substantial cytokine production (Fig 3E, 3F, 3G and 3H). Given that IFN-γ, a Th1-derived cytokine, and IL-17, a Th17-derived cytokine, enhance antifungal effector functions in macrophages and neutrophils [37–39], these findings demonstrate that mXJ104 effectively bridges *Candida* recognition with T-cell activation. This mechanism drives polyfunctional CD4^+^ and CD8^+^ T cell responses that generate critical antifungal effector cytokines.

One-step Protein A purification consistently yielded mXJ104 with over 95% monomer content as measured by SEC. After one week of incubation at 25°C, mXJ104 demonstrated excellent structural stability, maintaining consistent monomer percentages throughout the observation period (S4C Fig). These results indicate that mXJ104 possesses good thermal stability with minimal structural changes under the tested conditions.

In summary, mXJ104 displays high purity, excellent thermal stability, and specific functional activity, all of which are critical characteristics for further therapeutic development.

### mXJ104 enhances antifungal immunity in mice with candidemia

**Fig 4:**
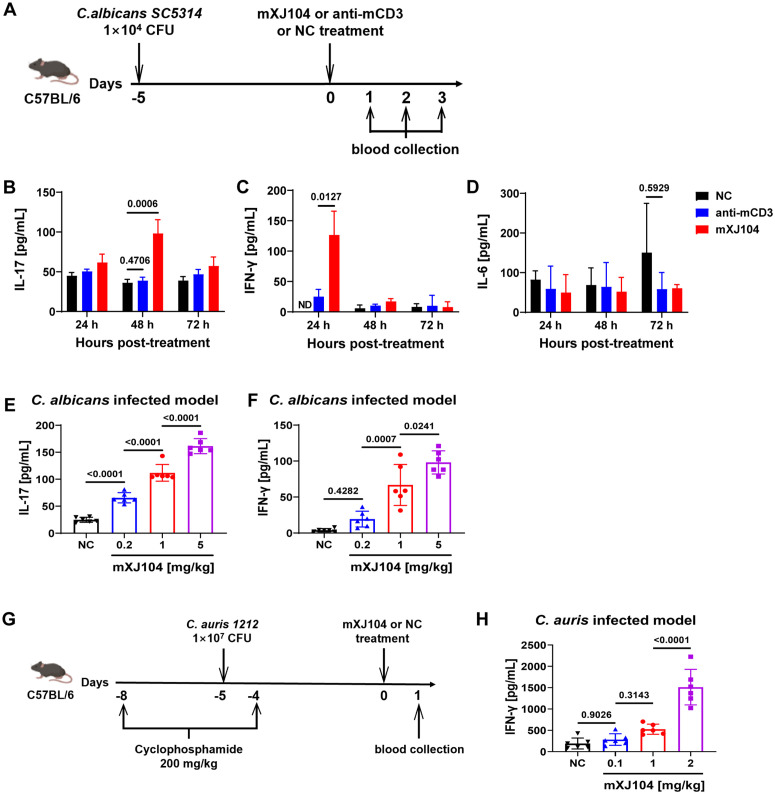
mXJ104 demonstrates significant immunomodulatory effects by enhancing immune responses in murine models of candidemia. (A) C57BL/6 mice were intravenously infected with *C. albicans SC5314* (1 × 10^4^ CFU, defined as day -5) and treated with mXJ104, anti-mCD3, NC on day 0, respectively. (B-D) Serum cytokines levels measured by ELISA at indicated time points; IL-17 (B), IFN-γ (C), and IL-6 (D). Mice received a 1 mg/kg antibody dose; n = 5 (24 h, 72 h), n = 6 (48 h). (E-F) Dose-response analysis of IL-17 (E, at 48 h post-treatment) and IFN-γ (F, at 24 h post-treatment) in serum from mXJ104-treated mice (n = 6). The dose of NC was 5 mg/kg. (G-H) C57BL/6 mice were intravenously infected with *C. auris 1212* (1 × 10^7^ CFU, day -5), and treated with mXJ104 or NC (2 mg/kg) on day 0, respectively (n = 6). Serum IFN-γ was measured at 24 h post-treatment. Data are means ± SD (n = 5 or 6) and are representative three independent experiments (B, C, D); Data are representative three independent experiments (E, F, H). NC, negative unrelated antibody; ND, no detection; h, hour. ****, *P* < 0.0001; ***, *P* < 0.001; *, *P* < 0.05; ns > 0.05; Two-way ANOVA with Dunnett’s multiple comparisons test (B, C, D); One-way ANOVA with Tukey’s multiple comparisons test (E, F, H).

To assess the immunomodulatory effects of mXJ104 *in vivo*, we administered mXJ104 intravenously to *C. albicans*-infected C57BL/6 mice and measured serum levels of IL-17 and IFN-γ (Fig 4A). mXJ104 treatment resulted in significantly elevated levels of both cytokines compared to anti-mCD3 or negative control (NC) antibody treatment. Peak IL-17 concentrations occurred at 48 hours post-treatment, whereas IFN-γ levels reached their maximum at 24 hours (Fig 4B and 4C). Notably, no detectable cytokine levels were observed in uninfected mice treated with mXJ104 (all measurements below the assay’s lower limit of detection), consistent with pathogen-dependent immunomodulation. Analysis of IL-6, a pro-inflammatory cytokine associated with cytokine storm syndrome (CRS) [40], revealed no significant differences between treatment groups (Fig 4D). Both IL-17 and IFN-γ levels showed positive correlations with mXJ104 dosage (Fig 4E and 4F), indicating dose-responsive immune modulation in *C. albicans*-infected mice. Comparable immunomodulatory effects were observed in a *C. auris 1212*-infected murine model (Fig 4G and 4H).

Together, these results demonstrate that mXJ104 enhances antifungal immunity in murine candidemia models by augmenting IFN-γ and IL-17 production, supporting further investigation of mXJ104’s protective efficacy in *Candida* infected models.

### mXJ104 displays a protective effect in mice with candidemia

**Fig 5:**
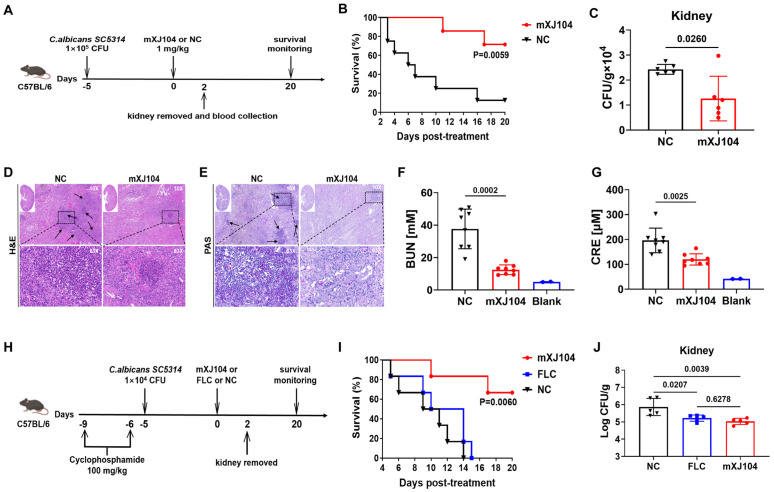
mXJ104 provides protective effects in murine models of *C. albicans*-induced candidemia. (A-G) Study in immunocompetent murine model. (A) Experimental timeline for infection and treatment in immunocompetent mice. (B) Survival of mice was monitored for 20 days (n = 8). (C) The fungal burden in the kidneys of mice at 48 hours post-treatment (n = 6). (D-E) Representative H&E (D) and PAS (E) staining of kidneys from infected mice after 48 h of treatment. Arrows indicate inflammatory cells influx and tissue necrosis (H&E staining) and *C. albicans* filaments in the tissues (PAS staining). (F-G) BUN (F) and CRE (G) levels in mice were determined at 48 h post-treatment (n = 8); Blood collected from naive mice without treatment was served as blank control (n = 2). (H-J) Study in immunosuppressed murine model. (H) Experimental timeline for infection and treatment in immunosuppressed murine model. (I) Survival of mice was monitored for 20 days (n = 6). (J) The fungal burden in the kidneys of mice at 48 hours post-treatment (n = 6). Data are representative of three independent experiments (B, C, F, G, I, J); Data are representative image of five mice (D, E). H&E staining, Hematoxylin and Eosin staining; PAS staining, Periodic acid-Schiff staining; BUN, blood urea nitrogen; CRE, creatinine; FLC, fluconazole; NC, negative control. **, *P* < 0.01; *, *P* < 0.05; ns > 0.05; Log-rank (Mantel-Cox) test (B, I); Kolmogorov-Smirnov nonparametric test (C, F, G); One-way ANOVA with Tukey’s multiple comparisons test (J).

IFN-γ, secreted by Th1 cells, enhances macrophage phagocytic activity against *Candida* pathogens and promotes dendritic cell maturation, subsequently activating both Th1 and Th17 cell responses. IL-17, produced by Th17 cells, mobilizes neutrophils and stimulates β-defensin production, both of which are essential for rapid infection control invasion sites [37–39]. Based on these established mechanisms and our experimental findings, we evaluated the protective efficacy of mXJ104 against invasive candidiasis. Candidemia is the most frequently diagnosed presentation of invasive candidiasis, with most cases being healthcare-associated [9]. To evaluate the protective efficacy of mXJ104, we established murine candidemia models using an isotype control antibody as NC (Fig 5A). In *C. albicans SC5314*-infected mice, mXJ104 treatment (1 mg/kg) significantly improved survival compared to the control group (*P*=0.0059; Fig 5B). We next examined mXJ104’s role in host defense by quantifying renal fungal burden and histopathology, as kidneys are primary target organs during disseminated infection. At 48 hours post-treatment, mXJ104-treated mice showed significantly lower renal fungal burdens versus the NC group (*P*=0.0260; Fig 5C). Histopathological analysis demonstrated reduced inflammatory infiltrates, tissue necrosis, and hyphal invasion in the kidneys of mXJ104-treated mice (Fig 5D and 5E). Furthermore, we measured blood urea nitrogen (BUN) and creatinine (CRE), established biomarkers of renal dysfunction. mXJ104 treatment significantly reduced BUN (*P*=0.0002) and CRE (*P*=0.0025) levels compared to those in mice with NC treatment (Fig 5F and 5G). Together, these results indicate that mXJ104 treatment significantly attenuates renal injury in murine candidemia models.

Given the high prevalence of invasive candidiasis in immunocompromised patients, we further evaluated the protective efficacy of mXJ104 in cyclophosphamide-induced immunosuppressed mice infected with **C. albicans* SC5314* (Fig 5H). mXJ104 treatment significantly improved survival rates compared to both the fluconazole (*P* = 0.0060) and NC groups (Fig 5I). Notably, renal fungal burdens were comparable between mXJ104- and fluconazole-treated mice (*P* = 0.6278; Fig 5J).

The global emergence of non-*albicans Candida* species and increasing resistance underscore the urgent need for novel broad-spectrum therapies [9]. As β-1,3-glucan—the target of mXJ104—is a conserved component of the *Candida* cell wall, we previously demonstrated the ability of mXJ104 to enhance immune responses in a *C. auris 1212*-infected murine model. To further investigate its therapeutic potential, we evaluated the protective efficacy of mXJ104 in enhancing host defenses against additional non-*albicans Candida* species infections. We found that treatment with mXJ104 significantly reduced renal fungal burdens in murine candidemia models caused by both *C. auris* and *C. tropicalis* (*P* = 0.0079; S5 Fig)*.*

Collectively, these *in vivo* results support our hypothesis that mXJ104 provides protection against invasive candidiasis caused by both *C. albicans* and non-*albicans Candida* species.

### mXJ104-mediated protection against invasive candidiasis requires T cell responses

**Fig 6:**
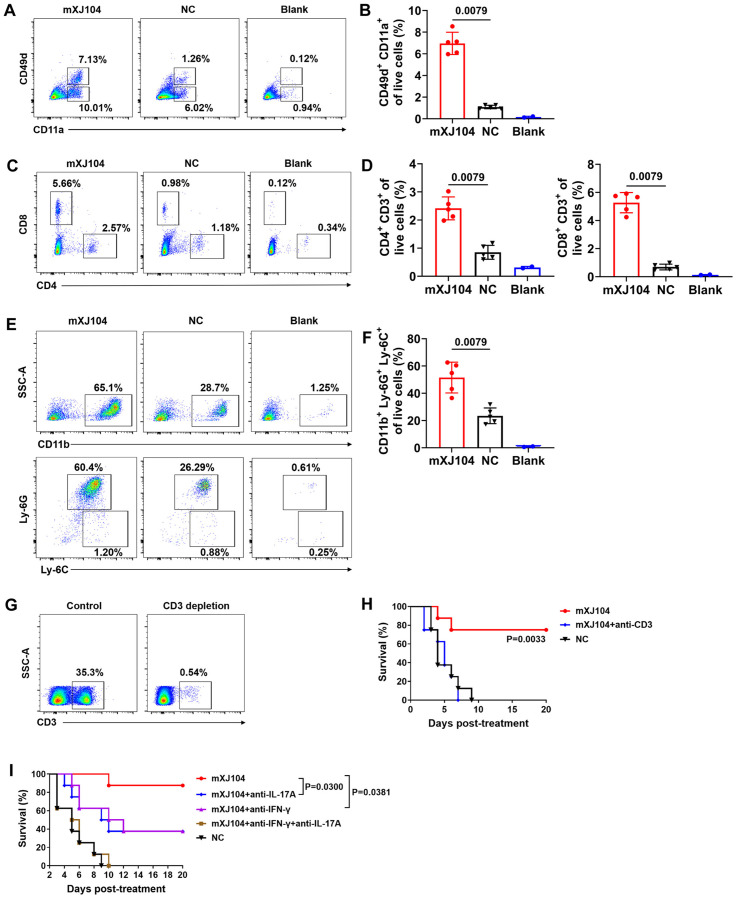
T cell response is required for the protective efficacy of mXJ104 in invasive candidiasis murine model. C57BL/6 mice were intravenously infected with *C. albicans SC5314* (1 × 10^5^ CFU, defined as day -5) and treated with mXJ104 or NC (1 mg/kg) on day 0, respectively. **(A)** Expression of CD11a and CD49d in kidney cells. Graph in (B) shows the frequency of CD49d^+^ CD11a^+^ cells. **(C-D)** T cells recruitment in kidney. CD4^+^ and CD8^+^ cells were gated on live cells **(C)**. Graphs in (D) shows the frequency of CD4^+^ (left panel) and CD8^+^ (right panel) cells. **(E-F)** The neutrophil infiltration in kidney. **(E)** CD11b^+^ cells were gated on live cells (top panel), then Ly-6G^+^ Ly-6C^+^ cells were gated on CD11b^+^ cells (bottom panel). Graph in (F) shows the frequency of CD11b^+^ Ly-6G^+^ Ly-6C^+^ cells. Kidneys were harvested at 48 hours post-treatment, and data are representative image of five mice (A, C, E) and are means ± SD (n = 5; B, D, **F)**; **, *P* < 0.01. Left panels, infected mice with mXJ104 treatment; middle panels, infected mice with NC treatment; right panels, naïve mice without treatment. **(G)** Flow cytometry demonstrating CD3^+^ T cell depletion; data are representative image of three mice. **(H)** Survival of infected mice treated with anti-CD3 depleting antibody (n = 8); **, *P* < 0.01 (mXJ104 + anti-CD3 versus mXJ104). **(I)** Survival of infected mice treated with neutralizing antibodies; *, *P* < 0.05 (mXJ104 + anti-IL-17A versus mXJ104; mXJ104 + anti-IFN-γ versus mXJ104). Data are representative of three independent experiments (H, **I)**. Kolmogorov-Smirnov nonparametric test (B, D, **F)**; Log-rank (Mantel-Cox) test (H, **I)**. NC, negative control.

To determine whether mXJ104 induces antigen-experienced T cell responses, we evaluated CD11a and CD49d expression on T cells in spleen and kidneys of *C. albicans*-infected mice treated with mXJ104 or NC. This methodological approach has been rigorously validated in studies of viral, *Plasmodium*, and *Listeria monocytogenes* infections [41–43]. In uninfected, untreated mice, CD49d^+^ CD11a^+^ cells constituted a minimal fraction (~0.12%) of renal leukocytes (Fig 6A). In contrast, by 48 hours post-treatment, mXJ104-treated infected mice exhibited significantly expanded populations of CD49d^+^ CD11a^+^ CD4^+^ and CD8^+^ T cells in both spleen and kidneys compared to NC-treated controls (Fig 6A and 6B; S6 Fig). These integrins are known mediators of leukocyte trafficking to inflammatory sites [42]. We subsequently quantified T cell recruitment to infected kidneys during systemic candidiasis. Consistent with integrin upregulation, mXJ104 treatment resulted in substantially higher proportions of renal CD4^+^ (2.57% vs. 1.18%) and CD8^+^ T cells (5.66% vs. 0.98%) compared to NC-treated mice at 48 hours post-treatment (Fig 6C and 6D). Parallel analysis of innate immune responses revealed enhanced neutrophil infiltration in mXJ104-treated mice. Renal CD11b^+^Ly-6C^+^Ly-6G^+^ neutrophil numbers were significantly elevated at 48 hours post-treatment, correlating with reduced fungal burden and improved renal function (as evidenced by decreased BUN and CRE levels) (Fig 6E and 6F).

To further investigate the mechanistic basis of mXJ104-mediated protection *in vivo*, we employed two functional disruption models. First, we assessed the role of CD3^+^ T cells by administering an anti-CD3 depleting antibody (InVivoMAb anti-mouse CD3ε), with depletion confirmed by flow cytometry (Fig 6G). Within 20 days, CD3^+^ T cell depletion significantly reduced survival in infected mice treated with mXJ104 (*P* = 0.0033, Fig 6H). We next evaluated the contribution of key cytokines using neutralizing antibodies against IL-17A and/or IFN-γ. Compared to mXJ104-treated mice receiving anti-IFN-γ (*P* = 0.0381), anti-IL-17A (*P* = 0.0300), or both neutralizing antibodies, infected treated with mXJ104 alone exhibited significantly higher survival rates (Fig 6I).

Taken together, these findings indicate that mXJ104-mediated protection involves T-cell activation, migration, cytokine production (IFN-γ and IL-17), and neutrophil recruitment to inflammatory sites.

### hXJ104 maintains specific interaction with target cells

**Fig 7:**
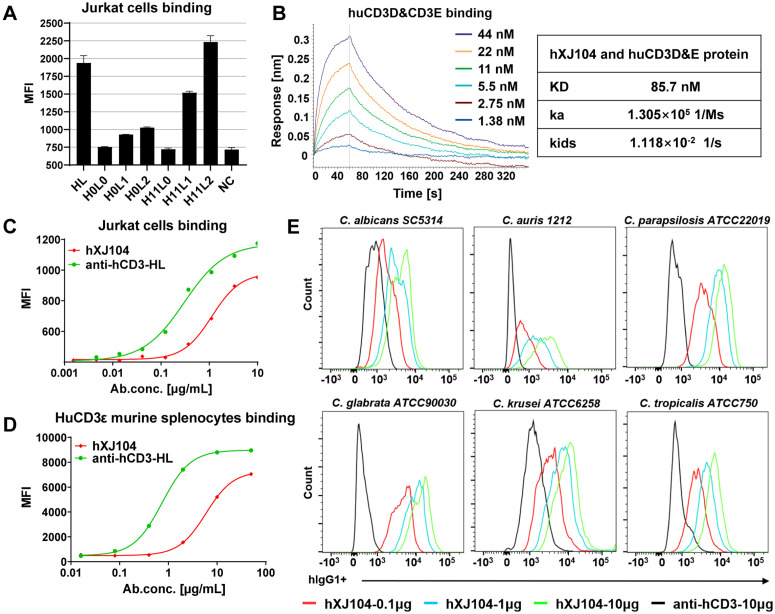
hXJ104 simultaneously binds to CD3-expressed cells and various pathogenic *Candida* species. (A) Affinities of anti-hCD3 variants to Jurkat cells. MFI of Jurkat cells incubated with antibodies were determined by flow cytometry. Data are means ± SD from triplicates of one representative experiment of three. (B) BLI analysis for interaction between hXJ104 and huCD3D&CD3E protein. KD, ka and kids values showed in the table. (C-E) Flow cytometry for hXJ104 binding to Jurkat cells (C), splenocytes from huCD3ε transgenic mice (D), and various clinically prevalent *Candida* species (E); Data are representative of three independent experiments (B, C, D, E); MFI, mean fluorescence intensity; BLI, bio-layer interferometry; KD, equilibrium dissociation constant; ka, association rate values; kd, dissociation rate values.

Based on the promising preclinical profile of mXJ104, we developed hXJ104, a humanized TCE targeting human CD3. For the CD3-targeting domain, we generated six humanized antibodies by grafting complementarity-determining regions (CDRs) from the murine anti-human CD3 antibody SP34 [44] onto human framework regions. Since CRS and other significant toxicities limit TCE applications [45], we pursued affinity optimization to reduce T-cell overactivation. Flow cytometric analysis of binding to human CD3-expressing Jurkat cells identified the anti-hCD3H11L1 variant as exhibiting optimal affinity (Fig 7A).

For hXJ104 production, we preserved the structural architecture of mXJ104 while replacing the murine CD3-binding domain with the anti-hCD3H11L1 Fab fragment. A low-affinity CD3-binding arm (KD ≈ 50–200 nM) may optimize tumor distribution by minimizing CD3-mediated plasma clearance and reducing accumulation in T-cell-rich tissues [46–48]. Biolayer interferometry (BLI) assays confirmed hXJ104’s specific binding to recombinant huCD3D&CD3E heterodimer (KD = 87.5 nM, Fig 7B), supporting its suitability for *in vivo* evaluation. Cellular binding assays revealed robust binding of hXJ104 to both Jurkat cells and splenocytes isolated from B-huCD3E transgenic mice (Fig 7C and 7D). The construct maintained broad-spectrum recognition of clinically relevant *Candida* species (Fig 7E), suggesting potential efficacy against diverse fungal pathogens. Accelerated stability testing showed maintained high purity (>95% monomer content) and antigen-binding capacity after one-week incubation at 25°C and 40°C (S8 Fig), meeting critical developability criteria for therapeutic antibodies.

These collective findings demonstrate successful retention of mXJ104’s favorable characteristics in the humanized derivative while achieving species cross-reactivity, supporting progression to formal efficacy and safety assessment.

### hXJ104 exhibits typical pharmacokinetic characteristics and well tolerability in cynomolgus

Although TCEs show therapeutic promise, their clinical applications remain limited by dose-dependent toxicities including CRS and neurotoxicity [45]. These safety considerations prompted rigorous evaluation of hXJ104 as an antifungal candidate. We conducted single-dose pharmacokinetic (PK) and tolerability studies in cynomolgus monkeys to assess its safety profiles.

**Fig 8:**
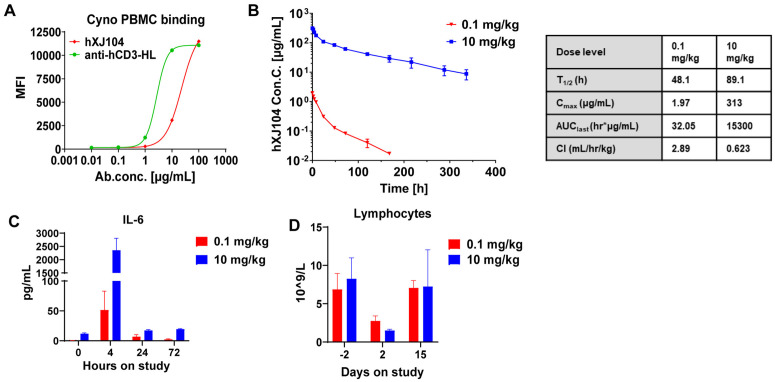
hXJ104 displays characteristic pharmacokinetic properties and well tolerability in cynomolgus monkeys. (A) Flow cytometry for hXJ104 binding to PBMCs isolated from cynomolgus. Anti-hCD3-HL served as control; Data are representative of three independent experiments. (B-D) Cynomolgus were dosed with slow intravenous infusion of hXJ104 at the dose of 0.1 mg/kg (n = 2) and 10 mg/kg (n = 2). Blood samples were collected at the indicated time points. (B) PK of hXJ104 in cynomolgus. The PK parameters presented in the table on the right. Blood markers of inflammation (IL-6, C), T cell activation (lymphocyte margination, D) were measured at the indicated time points. Data are means ± SD (n = 2) and are representative of three independent experiments (B, C, D). PBMCs, peripheral blood mononuclear cells; PK, Pharmacokinetic; IL-6, interleukin-6.

Flow cytometry confirmed cross-reactivity between hXJ104 and cynomolgus monkey peripheral blood mononuclear cells (PBMCs) (Fig 8A). PK analysis revealed dose-linear characteristics across tested doses, consistent with the behavior of a non-binding human IgG1 antibody (Fig 8B). Safety tests revealed two temporary effects common to this drug class, a brief rise in IL-6 levels that returned to baseline within 24 hours (Fig 8C), and a temporary decrease in lymphocyte counts that recovered within two weeks (Fig 8D). We carefully monitored the healthy parameters of the animals, including body weight, electrocardiogram readings, temperature, and feeding behavior, finding no treatment-related abnormalities.

Overall, hXJ104 demonstrated favorable safety profiles at doses up to 10 mg/kg in cynomolgus, with no severe side effects that would limit its clinical use.

### hXJ104 displays stronger activation effect *in vitro* and protective effect *in vivo*

**Fig 9:**
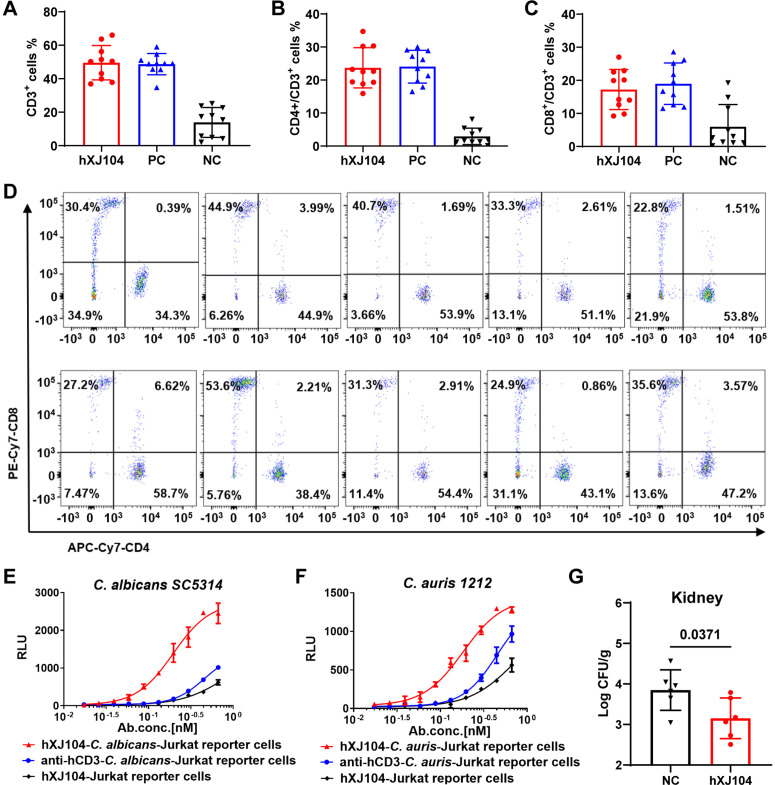
hXJ104 induces the activation of effector cells and protects the humanized mice from candidemia. (A-D) Cell-binding activity of hXJ104 to human peripheral blood cells analyzed by flow cytometry (n = 10). (A) Frequencies of cells reactive to hXJ104, PC, NC in human peripheral blood cells, respectively. Proportion of CD4^+^ cells (B) and CD8^+^ cells (C). Each symbol represented one sample. (D) Cells binding to hXJ104 encompassed both CD4^+^ and CD8^+^ T cells. Each image represented one sample. (E, F) Jurkat reporter cells activation assay. Jurkat reporter cells (5 × 10^4^ cells) were incubated with hXJ104 in the presence of 2 × 10^5^ cells *C. albicans SC5314* (E) or *C. auris 1212* (F). Luciferase substrate was added after 6 hours, then RLUs were measured. (G) C57BL/6 mice transferred with huCD3ε gene were infected with *C. albicans SC5314* (1 × 10^5^ CFU) on day -5, subsequently treated with hXJ104 or NC (1 mg/kg) on day 0 (n = 6). Fungal burdens of kidneys were quantified at 48 h after treatment. PC, positive control; NC, negative control; RLU, relative light unit. Data are representative of three independent experiments (A, B, C, D, G); Data are means ± SD (n = 2) and are representative of three independent experiments (E, F). *, *P* < 0.05, Kolmogorov-Smirnov nonparametric test (G).

To investigate the T-cell binding specificity of hXJ104, we performed flow cytometry analysis of peripheral blood cells from 10 healthy donors. Cells were labeled with either hXJ104 or an anti-CD3 positive control antibody. Quantitative analysis revealed that hXJ104 exhibited binding efficiency equivalent to that of the anti-CD3 positive control antibody in all donor samples (Fig 9A, 9B and 9C), verifying its specific interaction with CD3 subunits on T cells. Phenotypic analysis indicated that hXJ104-bound cells were primarily CD4^+^ and CD8^+^ T-cell subsets (Fig 9D and S9). The consistent binding pattern across donors suggests consistent target engagement in diverse populations.

We assessed the T-cell activation capacity of hXJ104 using a dual-cell coculture system containing luciferase-transduced Jurkat reporter cells (effectors) and either *C. albicans* or *C. auris* (targets). hXJ104 induced significantly stronger dose-dependent luciferase activity than the anti-hCD3 mAb when cocultured with either *Candida* species, while showing minimal activation in pathogen-free controls (Fig 9E and 9F). This pathogen-dependent activation profile recapitulated our prior observations with mXJ104, confirming that the activity of hXJ104 strictly depends on interaction with *Candida*.

To evaluate therapeutic potential, we used humanized CD3ε transgenic mice with systemic candidemia. hXJ104 treatment (1 mg/kg) significantly reduced renal fungal burdens in *C. albicans*-infected mice compared to negative controls (*P* = 0.0371; Fig 9G). This *in vivo* result aligns with our *in vitro* findings, supporting the protective efficacy of hXJ104 against candidemia.

## Discussion

Therapeutic challenges in invasive candidiasis highlight the urgent need for immunomodulatory strategies that enhance host antifungal defenses, complementing conventional antimicrobial approaches [49]. Building on the well-established foundations of T-cell-mediated antifungal immunity [19], we successfully engineered a novel TCE that activates and redirects T cells to sites of infection. Our TCE, named XJ104, establishes and maintains a specific and polyfunctional T-cell response *in vitro*, while *in vivo* it induces the generation of antigen-experienced T cells, promotes their migration to infected tissues, and stimulates the production of IFN-γ and IL-17 along with neutrophil recruitment at infection sites. Based on these findings, we further demonstrated that XJ104 confers protection in murine candidiasis models, with CD3^+^ T-cell responses and IL-17/IFN-γ production being essential for this protective efficacy. These results position XJ104 as a pathogen-directed immunotherapy that bridges antifungal recognition with immune activation, offering a promising therapeutic approach for invasive fungal diseases.

The therapeutic efficacy of TCEs is critically dependent on the structural architecture and functional valency of their antigen-binding domains (comprising anti-CD3 and tumor-associated antigen-targeting arms) [50,51]. Our design utilizes β-1,3-glucan, a conserved fungal cell wall component recognized by dectin-1, as the *Candida*-targeting moiety [22]. The CRD of dectin-1 shows high conservation across mammalian species, suggesting functional equivalence of this fungal-binding domain between species [30,52]. We employed the CRD of human dectin-1 to maintain the core binding specificity required for our proof-of-concept study, while aligning with our long-term objective of developing therapeutics for human applications. Several factors, including the multiplicity and diversity of protein-bound glycan structures, variable polarity of individual saccharide residues, and poor ionization efficiency, could interfere with mass spectrometer detection signals [53]. A previous study identified *N*-linked glycosylation at asparagine position 91 of dectin-1 [34]. Our data revealed that the 90–111aa region of dectin-1, which contains the glycosylation site, was undetectable by mass spectrometry. Therefore, we speculate that this region undergoes complex post-translational modifications resulting in abnormal protein structure. Notably, dectin-1(66–247aa)-Fc maintained specific binding to both *C. albicans* and β-1,3-glucan, indicating that while the structural abnormality is likely confined to the 90–111aa region, the CRD retains proper folding. This interpretation was further supported by the normal mass spectra, high purity, and preserved binding activity of both dectin-1(115–247aa)-Fc and dectin-1(119–247aa)-Fc, which lack the 90–111aa region. Through systematic analyses, we established dectin-1(119–247aa) as the optimal *Candida*-targeting module for TCE engineering.

To our knowledge, this study represents the first successful engineering of a PRR into a TCE for antifungal immunotherapy. The core mechanism of TCEs centers on T-cell activation, which was consistently demonstrated by our *in vitro* and *in vivo* findings with mXJ104. This molecule specifically activated T cells and induced effector cytokine production, particularly IL-17 and IFN-γ, both well-established mediators that recruit and activate innate immune cells to establish a protective microenvironment essential for pathogen control [15,54,55]. Notably, alongside these protective cytokines, we observed elevated IL-10 levels in our assays. Current evidence suggests that IL-10 plays conflicting roles during infection. While it suppresses inflammatory responses and mitigates tissue damage, it may also impair pathogen clearance and contribute to chronic disease. This context-dependent duality appears to be regulated by the initiating signals, cellular sources of IL-10, the dose and strain of the invading pathogen, and the target cells of IL-10 [56]. Given these complexities, the functional impact of mXJ104-induced IL-10 in invasive candidiasis warrants further investigation.

Previous studies have demonstrated that BiTEs (a class of TCEs) mediate target cell death primarily through antigen-experienced T cells, which differentiate into effector memory T cells (T_em_) after activation [50]. Consistent with these findings, hepatitis B virus-specific TCE has been shown to induce memory phenotypes in T cells [25]. Our *in vivo* data demonstrated that mXJ104 increases the proportion of CD49d^hi^CD11a^hi^ T cells in the kidneys and spleen of *C. albicans*-infected mice. This surface marker combination has been widely validated for identifying antigen-experienced T-cell responses to various pathogens, including viruses, *Plasmodium spp.*, *Ehrlichia muris* and *Listeria monocytogenes* [41–43]. Noah S. Butler et al. specifically confirmed the fidelity and durability of the CD49d^hi^CD11a^hi^ phenotype for identifying *Plasmodium*-specific effector and memory CD4^+^ T cells [43]. Collectively, these findings suggest that mXJ104 may promote the differentiation of activated T cells into memory subsets. This potential is particularly relevant for invasive candidiasis patients, who remain immunocompromised post-treatment and face high recurrence risks. The ability to induce memory T-cell responses could thus enhance the clinical value of XJ104, warranting further investigation.

The kidney serves as the primary target in disseminated candidiasis, yet exhibits impaired recruitment of antigen-specific CD4^+^ T cells [57]. Notably, our *in vivo* data demonstrate that mXJ104 enhances the recruitment of CD4^+^ T cells, albeit at lower levels than CD8^+^ T cells. Artificially restoring CD4^+^ T-cell responses in the kidney retains antifungal efficacy [57]. Correlative findings include increased neutrophil infiltration, reduced fungal burden, and improved renal function (evidenced by histopathology and serum BUN/CRE levels), collectively indicating mXJ104-mediated infection control in the kidney.

A proof-of-concept clinical trial demonstrated that recombinant IFN-γ administration improves immunological parameters in patients with systemic candidiasis [15]. While IL-17A administration protects antibiotic-exposed mice from invasive candidiasis [58,59]. These preclinical and clinical findings support the translational potential of mXJ104, which induces both IL-17 and IFN-γ production *in vitro* and *in vivo*, and confers protection in murine candidemia models. Mechanistically, protective efficacy depends on T-cell activation and effector functions, as evidenced by substantially diminished efficacy in CD3^+^ T-cell-depleted mice and following IL-17A/IFN-γ neutralization. Notably, broad-spectrum antibiotics impair gut immunity mediated by IL-17A [58], and both broad-spectrum antibiotics and IL-17 inhibitors promote commensal *C. albicans* overgrowth, facilitating bloodstream invasion upon barrier disruption [1,13,60–62]. However, the inability of laboratory mice to sustain natural *C. albicans* colonization [63] precluded evaluation of mXJ104-induced IL-17 effects on fungal colonization, a limitation requiring investigation in gastrointestinal colonization models.

While our study primarily focuses on *C. albicans*, non-*albicans Candida* infections continue to increase, with multidrug-resistant species, being of particular concern [9]. mXJ104 demonstrated protective efficacy against *C. auris* and *C. tropicalis* infections in mice, while hXJ104 induced comparable Jurkat reporter cell activation across six *Candida* species *in vitro*, collectively suggesting the broad-spectrum potential of XJ104. Two other dectin-1-based immunotherapies targeting β-1,3-glucan, dectin-Fc [64] and dectin-1-CAR-T (D-CAR-T) cells [65], could control *Pneumocystis carinii* and *Aspergillus fumigatus* infections, respectively. These findings imply that the broad spectrum of XJ104 may extend to other β-1,3-glucan-rich fungal pathogens beyond *Candida*.

Critically, mXJ104 maintained protective efficacy in immunosuppressed models, which is a key translational advantage given invasive candidiasis predominance in immunocompromised patients [1]. Surprisingly, while mXJ104- and fluconazole-treated mice showed comparable renal fungal burdens at 48 hours post-treatment, mXJ104-treated mice had greater survival within 20 days. These results suggest that although fluconazole reduces fungal load, immunocompromised hosts remain susceptible to *Candida* infections with poor prognosis [7–9], whereas mXJ104, as an immunotherapeutic approach, restores immune function and improves outcomes. Notably, while mXJ104 targets β-1,3-glucan, it does not directly disrupt fungal cell wall integrity or exert fungicidal effects. Conventional antifungal drugs, despite direct microbicidal activity, often fail to achieve favorable clinical outcomes due to the impaired host immune response. Additionally, the growing problem of drug resistance further restricts treatment options [1,20]. Thus, combining immunotherapy with traditional antifungals may optimize infection control and clinical outcomes.

Immunotherapy, particularly TCEs, is a revolutionary approach for cancer treatment, yet its therapeutic potential is also limited by significant toxicities, such as CRS, which are directly associated with the induction of potent immune effector responses [66]. A key strategy to mitigate CRS is to limit T-cell activation through molecular strategies that reduce TCE affinity for CD3 [45]. Notably, comparative analyses suggest low-affinity anti-CD3 variants may offer clinical advantages over their high-affinity counterparts, underscoring the critical balance required between target affinity, biological potency, and toxicity profiles [46,67,68]. For hXJ104, we selected a moderate-affinity anti-humanCD3 Fab as the T-cell-binding arm, which demonstrated favorable safety in cynomolgus at 10 mg/kg. The molecular design further incorporates an Fc domain with L234A/L235A double-mutation [36] and a bivalent 2 + 2 format [69]. Importantly, the *Candida-* and β-1,3-glucan-dependent immune activation profile of XJ104 may further reduce toxicity risks, including CRS and neurotoxicity. In contrast to most tumor antigens that are also expressed on normal cells, XJ104 specifically targets β-1,3-glucan, a polysaccharide absent in mammalian cells, suggesting minimal off-target toxicity risk. While the B-huCD3E transgenic humanized mouse model demonstrated the efficacy of hXJ104, it should be noted that humanized mouse models may incompletely recapitulate the immunological complexity of human *Candida* infections and affect toxicity predictability. While antitumor TCEs typically employ step-up dosing over 21- to 28-day cycles to mitigate CRS [45,51], this approach may be incompatible with invasive candidiasis due to its rapid progression [9]. Combining XJ104 with conventional antifungals could represent an optimized strategy to achieve rapid pathogen clearance while reducing dose-dependent toxicity and resistance. Clinical data further support CRS management through glucocorticoid premedication, blocking antibodies administration, or subcutaneous delivery [51]. To accelerate clinical translation, future studies should establish preclinical models to evaluate XJ104-antifungal synergism, optimal dosing regimens, and administration routes.

In summary, our study demonstrated that the T-cell engager targeting β-1,3-glucan, a major fungal cell wall component, can effectively activate T cells to control *Candida* infections. These findings support a promising and feasible therapeutic strategy for invasive candidiasis treatment. Nevertheless, the therapeutic potential of this approach for mucosal candidiasis and the potential synergies with standard antifungal therapy require further investigation. Collectively, these results provide a strong rationale for developing first-in-class antifungal agents with this mechanism of action.

## Materials and methods

### Ethics statement

All mouse experiment procedures were performed in accordance with the Regulations for the Administration of Affairs Concerning Experimental Animals approved by the State Council of People’s Republic of China. The protocol was approved by the Institutional Animal Care and Use Committee of Tongji University (Permit Number: TJAA08020101).

Cynomolgus monkey safety and pharmacokinetic (PK) studies were entrusted to JOINN (Suzhou, China), an institution certified by Association for Assessment and Accreditation of Laboratory Animal Care International (AAALAC International), following the Guide for the Care and Use of Laboratory Animals (The Guide) and Animal Welfare Regulations (AWR). The protocols were approved by Institutional Animal Care and Use Committee (IACUC) (Permit Number: S-ACU23–0635, S-ACU23–0819).

Human peripheral whole blood was collected after obtaining written consent from all healthy donors. This study was approved by Ethics Committee for Biomedical Research Involving Human Subjects of Tongji University (Permit Number: 2023tjdxsy047).

### Mice

Female C57BL/6 mice (7–8-week-old, weighing 18-20g) were obtained from the Shanghai Laboratory Animal Center of the Chinese Academy of Sciences (Shanghai, China). Female C57BL/6-*Cd3e*^*tm2(CD3E)Bcgen*^/Bcgen (B-huCD3E) mice were purchased from Biocytogen (Beijing, China).

### Cell lines, reagents and antibodies

Jurkat cells (Clone E6-1) were purchased for Cell Bank of Chinese Academy of Sciences. Jurkat cells stably transfected with NFAT-luciferase reporter gene (Jurkat-NFAT-Luc Effector cells) was purchased from Vazyme, Nanjing of China. Both cells were cultured in RPMI1640 medium plus 10% (vol/vol) heat-inactivated fetal bovine serum (FBS) at 37°C with 5% CO_2_.

The ELISA kits for IL-10, IL-17, IFN-γ, IL-2, IL-6 detection were purchased from Invitrogen. Bio-Lite Plus Luciferase Assay System was purchased from Vazyme. Recombinant Human CD3D & CD3E Heterodimer Protein (ECD, His Tag) and Recombinant Mouse CD3D & CD3E Heterodimer Protein (Flag & His Tag) were purchased from SinoBiological. Urea Nitrogen Assay Kit and Amplex Red Creatinine Assay Kit were purchased from Beyotime. FITC anti-huIgG Fc (Clone M1310G05), APC/Cyanine7 anti-mouseCD3 (Clone 17A2), PE anti-mouseCD4 (Clone GK1.5), APC anti-mouseCD8 (Clone 53-6.7), FITC anti-mouseCD69 (Clone H1.2F3), FITC anti-mouseCD25 (Clone 3C7), APC anti-mouse/human CD11b (Clone M1/70), FITC anti-mouse Ly-6C (Clone HK1.4), PE anti-mouse Ly-6G (Clone 1A8), FITC anti-mouse CD11a (Clone M17/4) and PerCP/Cyanine5.5 anti-mouse CD49d (Clone R1-2) were purchased from Biolegend. APC anti-CD4 (Clone SK3), PE-Cy7 anti-CD8 (Clone SK1), PerCP anti-CD3 (Clone SK7) were purchased from BD Bioscience. Alexa Fluor 647 AffiniPure F(ab’)₂ Fragment Goat Anti-Human IgG, Fcγ fragment specific were purchased from Jackon Immuno Research. Goat anti-Human IgG Fc Cross-Adsorbed Secondary Antibody, HRP was purchased from Thermo Fisher. InVivoMAb anti mouse CD3 (Clone 145-2C11), InVivoMAb anti mouse IFN-γ (Clone XMG1.2) and InVivoMAb anti-mouse/rat IL-17A (Clone 17F3) was purchased from BioXCell.

### *Candida spp.* strains and growth conditions

All standard reference *Candida* strains were obtained from the BeNa Culture Collection (BNCC). *C. auris 1212* was isolated from a clinical specimen at Tenth People’s Hospital of Shanghai, China. *Mnn10* null mutant strain (*Mnn10*^*Δ/Δ*^) was generated by deleting the entire open reading frame of *MNN10* through homologous recombination of auxotrophic markers HIS1 and LEU2 using a fusion-PCR-based strategy as previous study [35]. All *Candida* strains were routinely cultured on sabouraud dextrose agar (SDA) plates (1% peptone, 4% dextrose, and 1.8% agar) for isolation of individual colonies and cultured in yeast extract-peptone-dextrose (YPD) broth (1% yeast extract, 2% peptone, and 2% dextrose) at 30 °C (37 °C for *C. auris 1212*) in a shaking incubator.

### Production of dectin-1-Fc fusion proteins and T cell engagers

All dectin-1-Fc fusion proteins and TCEs were expressed using the Expi293 Expression System following the manufacturer’s instructions. Briefly, recombinant expression plasmids encoding each protein were transformed into Expi293 cells. Transfected cells were cultured at 37°C with 8% CO_2_ for 6 days in a humidified incubator. Culture supernatants were harvested and subjected to protein purification via Protein-A affinity chromatography.

### Flow cytometry analysis of dectin-1 binding

All binding assays were performed with exponentially growing *Candida* cells (2.5 × 10^6^ CFU) fixed in 4% paraformaldehyde at room temperature (RT) for 45 minutes. Fixed cells were incubated for 45 minutes at RT with dectin-1(66–247aa)-Fc (0.1 or 25 μg for *C. albicans*; 10 μg for comparative analysis with other truncations), dectin-1(115–247aa)-Fc (10 μg), dectin-1(119–247aa)-Fc (10 μg), mXJ104 (1–20 μg for C. albicans; 0.1-10 μg for non-albicans species), hXJ104 (0.1-10 μg), or negative control antibody (matched to experimental doses), respectively. After PBS containing 1% FBS washes, cells were stained with FITC anti-huIgG Fc (30 minutes, RT, dark) and analyzed by BD FACSVerse flow cytometry and FlowJo 10.8.1.

### Flow cytometry analysis of anti-CD3 binding

Wild-type murine splenocytes (1 × 10^6^ cells) were isolated from female C57BL/6 mice (7–8-week-old, weighing 18-20g) and incubated with anti-mCD3 (1 μg), a dilution series of mXJ104 or negative control antibody (1 μg), followed by staining with FITC anti-human IgG Fc. Fresh cells incubated with APC/Cyanine7 anti-mouseCD3 served as the positive control. The stained cells were analyzed by BD FACSVerse flow cytometry and FlowJo 10.8.1.

Jurkat cells (5 × 10^6^ cells) were incubated with anti-hCD3-HL, the humanized variants or a negative control antibody (5 μg) or a dilution series of hXJ104 or anti-hCD3-HL, following by staining with FITC anti-human IgG Fc and analysis by Intellicyt iQue3 flow cytometry and GraphPad Prism 9.5.

HuCD3ε murine splenocytes (1 × 10^6^ cells) were isolated from B-huCD3E humanized mice and incubated with a dilution series of anti-hCD3-HL or hXJ104. Cells were then stained with Alexa Fluor 647 AffiniPure F(ab’)₂ Fragment Goat Anti-Human IgG, Fcγ fragment specific, and analyzed by Attune NxT Flow Cytometer and GraphPad Prism 9.5.

Human peripheral blood (50 μL) was obtained from healthy volunteers and incubated with 5 μg hXJ104, PerCP-anti-CD3, or a negative control antibody, followed by staining with APC-anti-CD4 and PE-Cy7-anti-CD8. Then, cells were stained with FITC anti-human IgG Fc and analyzed by BD FACSVerse flow cytometry and FlowJo 10.8.1.

Cynomolgus PBMCs (1.8 × 10^6^ cells) were isolated by density gradient centrifugation over Ficoll-Paque-Plus from cynomolgus peripheral blood and incubated with a dilution series of anti-hCD3-HL or hXJ104. Then, cells were stained with PE-anti-human IgG Fc and analyzed by BD FACSVerse flow cytometry and GraphPad Prism 9.5.

### Enzyme-linked immunosorbent assay (ELISA)

Polysaccharides (Zymosan A, Mannan, Astragalus Polysacharin, α-1,6-glucan) were coated onto 96-wells plates (Thermo Scientific, 442404) at 2.5 μg/well overnight at 4°C, then blocked with PBS containing 5% BSA for 1 hour at RT. Series dilutions of dectin-1(66–247aa)-Fc, dectin-1(115–247aa)-Fc, dectin-1(119–247aa)-Fc, XJ104 or a negative control antibody were added and incubated for 1 hour at 37°C. After washing with PBS containing 0.05% tween-20 (PBST), plates were incubated with goat anti-human IgG Fc-HRP (1:10,000 dilution) for 1 hour at RT. Following additional washes, TMB was added for 15 minutes, and reactions were stopped with 1 M H_3_PO_4_. Absorbance was measured at 450 nm immediately. Data were analyzed using GraphPad Prism 9.5, and nonlinear regression analysis was performed to calculate the half-maximal binding concentration.

### Assessment of T cell activation *in vitro*

5 days prior to splenocyte isolation, female C57BL/6 mice were intravenously injected with 1 × 10^4^ CFU of *C. albicans SC5314*. Cell culture plates were coated with 10 μg/mL zymosan A at 4°C overnight, followed by removal of unbound zymosan A. Murine splenocytes (1 × 10^5^ cells/well) were cultured with serial dilutions of mXJ104 or anti-mCD3 (1nM) for 20 hours. Cells were stained with APC/Cy7 anti-mouseCD3, PE anti-mouseCD4, APC anti-mouseCD8, FITC anti-mouseCD69 or FITC anti-mouseCD25 for flow cytometric analysis of activation markers (BD FACSVerse; FlowJo 10.8.1). For cytokine profiling, supernatants were collected after 48 hours (IFN-γ, IL-2, IL-10) or 168 hours (IL-17) and analyzed by ELISA.

### Assessment of immune response *in vivo*

All infection and treatment timepoints were standardized to day -5 and day 0, respectively. Immunocompetent female C57BL/6 mice were injected with 1 × 10^4^ CFU of *C. albicans SC5314* intravenously on day -5, followed by treatment with mXJ104 (0.2, 1, 5 mg/kg), anti-mCD3 (1 mg/kg) and negative control antibody (NC; 1, 5 mg/kg) on day 0. Blood was collected at 24, 48, 72 hours post-treatment. For the *C. auris 1212* infection model, mice were rendered immunocompromised by intraperitoneal cyclophosphamide (200 mg/kg) administration on days -8 and -4, followed by infection with *C. auris 1212* (1 × 10^7^ CFU) on day -5. Treatment with mXJ104 (0.1, 1, 2 mg/kg) or NC (2 mg/kg) occurred on day 0, with blood collection 24 hours later. Serum concentrations of interleukin-17 (IL-17), interferon-gamma (IFN-γ) and IL-6 were quantified using ELISA kits.

### Assessment of protective effect *in vivo*

To evaluate the protective efficacy of XJ104, five murine candidemia models were established. All infection and treatment timepoints were standardized to day -5 and day 0, respectively.

Immunocompetent *C. albicans* model: Mice were intravenously infected with *C. albicans SC5314 (*1 × 10^5^ CFU) on day -5. Five days post-infection (day 0), mice were received mXJ104 or NC (1 mg/kg). For survival analysis (n = 8), mice were monitored for 20 days with ad libitum access to food/water. In the fungal burden (n = 6) and histopathology (n = 3) examination experiment, kidneys were removed and examined at 48 hours post-treatment (day 2). Blood was collected at 48 hours post-treatment. The levels of blood urea nitrogen (BUN) and creatinine (CRE) were measured by Urea Nitrogen Assay Kit and Amplex Red Creatinine Assay Kit. For *in vivo* depletion of CD3 expressing cells, mice were infected i.v. every third day with 50μg of InVivoMAb anti mouse CD3 (n = 8). For *in vivo* blocking of IFN-γ or/and IL-17, mice were infected i.v. every third day with 50μg InVivoMAb anti mouse IFN-γ, InVivoMAb anti-mouse/rat IL-17A, or both neutralizing antibodies (n = 8).

Cyclophosphamide-induced immunosuppressed *C. albicans* model: Cyclophosphamide (100 mg/kg) was administered intraperitoneally on day -9 and -5. Mice were infected with *C. albicans SC5314 (*1 × 10^4^ CFU, i.v.) on day -5, then randomized into three groups receiving NC (1 mg/kg, i.v.), fluconazole (0.2 mg/kg, i.p.), mXJ104 (1 mg/kg, i.v.) on day 0, followed by 20-days survival monitoring (n = 6). At 48 hours post-treatment (day 2), the kidneys were removed, and then homogenized in PBS buffer to determine fungal burden (n = 5).

Cyclophosphamide-induced immunosuppressed *C. auris* model: Cyclophosphamide (200 mg/kg, i.p.) was administered on day -8, -4. Mice were infected with *C. auris 1212* (3 × 10^7^ CFU, i.v.) on day -5. On day 0, mice received NC (1 mg/kg, i.v.), or mXJ104 (1 mg/kg, i.v.). At 48 hours post-treatment (day 2), the kidneys were removed, and then homogenized in PBS buffer to determine fungal burden (n = 5).

Immunocompetent *C. tropicalis* model: Mice were intravenously infected with *C. tropicalis ATCC750* (5 × 10^5^ CFU) on day -5. 5 days post-infection (day 0), mice were received mXJ104 or NC (1 mg/kg). In the fungal burden (n = 5) experiment, kidneys were harvested and examined at 48 hours post-treatment (day 2).

Humanized mouse model: B-huCD3E transgenic mice (huCD3ε^+^, C57BL/6 background) were intravenously infected with *C. albicans SC5314* (1 × 10^5^ CFU) on day -5. On day 0, mice received hXJ104 or NC (1 mg/kg). All mice were euthanized 48 hours post-treatment (day 2) for kidney fungal burden assessment (n = 6).

### Assessment of antigen-experienced T cells

Mice were intravenously infected with *C. albicans SC5314* (1 × 10^5^ CFU) on day -5. 5 days post-infection (day 0), mice were received mXJ104 or NC (1 mg/kg). Kidneys and spleen were removed at 48 hours post-treatment. Kidneys were digested and passed through a-70-mm filter, washed and centrifuged in a 40%/70% percoll gradient for leukocyte isolation. Leukocytes isolated from kidneys and spleen respectively were stained with FITC anti-mouse CD11a, PerCP/Cyanine5.5 anti-mouse CD49d, APC/Cy7 anti-mouseCD3, PE anti-mouseCD4 and APC anti-mouseCD8, then analyzed by BD FACSVerse flow cytometry and FlowJo 10.8.1.

### Assessment of neutrophils recruitment in the kidney

Mice were intravenously infected with *C. albicans SC5314 (*1 × 10^5^ CFU) on day -5. 5 days post-infection (day 0), mice were received mXJ104 or NC (1 mg/kg). The leukocytes were isolated from kidneys at 48 hours post-treatment and stained with APC anti-mouse/human CD11b, FITC anti-mouse Ly-6C, and PE anti-mouse Ly-6G, then analyzed by BD FACSVerse flow cytometry and FlowJo 10.8.1.

### Biolayer interferometry assay (BLI)

The recombinant huCD3D&CD3E protein was serially diluted in PBST to the different concentrations. hXJ104 was also diluted to 20 μg/mL with PBST. Octet RED384 (Sartorius) combined with Octet ProA Biosensors (Sartorius) was used for BLI analysis. Each cycle involved hXJ104 loading, recombinant huCD3D&CD3E protein association, dissociation, elution and regeneration. The 1:1 binding model in software were used to calculate the equilibrium dissociation constant KD, which was defined as the ratio of kdis/ka.

### Cynomolgus monkey safety and pharmacokinetic (PK) study

Two female and two male cynomolgus monkeys, with weights at the initiation of dosing ranging from 2-5 kg, were assigned to 2 groups. Both groups included one female and one male animal. Animals received hXJ104 at a dosage of 0.1 or 10 mg/kg via intravenous infusion. Animals were monitored twice daily for any abnormalities and signs of pain or distress.

Cynomolgus monkey blood was collected at various times throughout the study. Serum PK samples were analyzed by ELISA assay. Serum concentration-time profiles were used to estimate the following PK parameters using non-compartmental analysis (Phoenix 8.1). IL-6 and lymphocytes were determined by commercially available kits.

### Jurkat reporter cells activity assay

*Candida* species cells were adjusted to a density to 8 × 10^6^ cells/mL using complete 1640 (RPMI 1640 medium containing 10% FBS), then inactivated at 65°C for 90 minutes and seeded into 96-well cell culture plate with 2 × 10^5^ cells/25μL/well. Jurkat-NFAT-Luc Effector cells as reporter cells were seeded with 5 × 10^4^ cells/25μL/well. A dilution series of hXJ104 from 0.667 to 0.017 nM (25μL/well) was co-cultured with *Candida spp.* cells and Jurkat reporter cells at 37°C, 5% CO_2_ for 6 hours. The luciferase substrate was added and incubated for 10 minutes. Luminescence values were read on SpectraMax i3x reader. Data were analyzed using GraphPad Prism 9.5, and nonlinear regression analysis was performed to fit the curves.

### Statistical analysis

At least three biological replicates were performed for all experiments unless otherwise indicated. Log-rank (Mantel-Cox) test was used for survival data analysis. Two-way analysis of variance with Dunnett’s multiple comparisons test was used for analysis of multiple groups with multifactorial; One-way analysis of variance with Tukey’s multiple comparisons test was used for analysis of multiple groups with single-factor. For analysis of two groups, the Kolmogorov-Smirnov nonparametric test was used. Exact p-value was shown in the figures, and statistical significance was set at a *P*-value in the figure legend as: ****, *P* < 0.0001; ***, *P* < 0.001; **, *P* < 0.01; *, *P* < 0.05; ns > 0.05.

## Supporting information

S1 Fig The 119–247aa region of dectin-1 exhibits the highest stability. (A) Schematic depiction of dectin-1-Fc fusion protein. (B-D) Mass spectrometry analysis of dectin-1(66–247aa)-Fc peptide fragments spanning 72–87aa (B), 90–111aa (C), and 112–129aa (D). (E-G) Molecular weights of dectin-1(66–247aa)-Fc (E), dectin-1(115–247aa)-Fc (F), and dectin-1(119–247aa)-Fc (G) were analyzed by mass spectrometry. (H) Non-reducing SDS-PAGE of dectin-1-Fc fusion proteins. Data are representative of three independent experiments. SDS-PAGE, sodium dodecyl sulfate polyacrylamide gel electrophoresis.
(TIF)

S2 Fig The anti-mouseCD3 monoclonal antibody demonstrates specific binding to mouseCD3. (A) ELISA assay for anti-mouseCD3 binding to mouseCD3D&CD3E protein. Data are means ± SD (n = 2) and are representative of three independent experiments. (B) Representative images analysed by flow cytometry for detection of anti-mouseCD3 to murine splenocytes. Data are representative of three independent experiments; Commercialized APC/Cyanine7 anti-mouseCD3 antibody served as positive control; isotype unrelated antibody served as a negative control; ELISA, enzyme-linked immunosorbent assay.
(TIF)

S3 Fig L234A/L235A double mutant abrogates FcγR binding activity of mXJ104. BLI analysis for the binding between mXJ104 and FcγRI (A), FcγRIIa (B), FcγRIIb (C) and FcγRIIIa (D). L234/L235 (left), mXJ104 without the double mutant; A234/A235 (right), mXJ104 with the double mutant. Data are representative of three independent experiments. BLI, Bio-Layer Interferometry.
(TIF)

S4 Fig mXJ104 was produced with a robust and stable structural conformation. (A-B) Molecular weight of anti-mCD3-dectin-1(119–247aa) (mXJ104), as confirmed by mass spectrometer (A) and SDS-PAGE (B). (C) Thermal stability analysis of mXJ104 by SEC. Data are representative of three independent experiments (A, B, C). SDS-PAGE, sodium dodecyl sulfate polyacrylamide gel electrophoresis; SEC, size exclusion chromatography.
(TIF)

S5 Fig mXJ104 demonstrates broad-spectrum protective efficacy against candidemia caused by non-*albicans Candida* species. C57BL/6 mice were intravenously infected with *C. auris 1212* (3 × 10^7^ CFU) and *C. tropicalis ATCC750* (1 × 10^6^ CFU), and treated with mXJ104 or negative control antibody (1 mg/kg). Quantification of the fungal burden in kidneys at 48 hours post-treatment. The kidney fungal burden of mice infected with *C. auris 1212* (A) or *C. tropicalis ATCC750* (B), n = 5. Data are representative of three independent experiments (A, B); **, *P* < 0.01, Kolmogorov-Smirnov nonparametric test (A, B).
(TIF)

S6 Fig mXJ104 increases the proportion of antigen-experienced T lymphocytes. C57BL/6 mice were intravenously infected with *C. albicans SC5314* (1 × 10^5^ CFU, defined as day -5) and treated with mXJ104 or NC on day 0, respectively. (A) Representive CD11a and CD49d expression on CD4+ (Top panel) and CD8+ (bottom panel) CD3+ T cells in spleen from infected mice treated with mXJ104 (left panel) or NC (right panel). (B) Frequency of CD11a+ CD49d+ cells in kidneys from infected mice treated with mXJ104. (C) Frequency of CD8+ or CD4+ CD3+ T cells in CD11a+ CD49d+ (left panel) or CD11a+ CD49d^-^ (right panel) cells. Spleens and kidneys were harvested at 48 hours post-treatment, and data are representative image of five mice (A, B, C).
(TIF)

S7 Fig Gating strategy for flow cytometry analyses in figures 6A, 6C and 6E. Single cells were first gated from all events obtained from kidneys of mXJ104-treated infected mice, followed by the selection of live cells from the single-cell gate. (A) CD49d ⁺ CD11a⁺ cells were gated from the live cells. This population was confirmed to be CD3⁺ and composed of CD4⁺ and CD8 ⁺ T cells. (B) From live cells, CD4+ and CD8+ cells were gated and confirmed to be CD3+ T cells. (C) CD11b⁺ cells were gated from the live cells. This population was confirmed to consist predominantly of Ly-6G ⁺ Ly-6C⁺ cells.
(TIF)

S8 Fig hXJ104 demonstrates exceptional thermal stability. hXJ104 were left at 25°C or 40°C for 1 week with a concentration of 5 mg/mL. (A-B) Purity assessment of mXJ104 stability samples by SEC (A) and NR-CE (B). Data are representative of three independent experiments. (C) The zymosan A-binding activity of mXJ104 stability samples was evaluated by ELISA, Data are means ± SD (n = 2) and are representative of three independent experiments. SEC, size-exclusion chromatography; NR-CE, non-reduced capillary electrophoresis; ELISA, enzyme-linked immunosorbent assay.
(TIF)

S9 Fig hXJ104 could bind to human CD3+ T cells. Schematic of flow cytometry analysis from representative sample (n = 10). Firstly, live cells were gated, and then cells reactive to hXJ104 were gated. Subsequently, CD4+ and CD8+ cell subsets were also gated in hXJ104 reactive cells and all live cells.
(TIF)

S1 Table All original data used to build the graphs, including values to build figures 1C; 2A, 2D; 3C, 3D, 3E, 3F, 3G and 3H; 4B, 4C, 4D, 4E, 4F and 4H; 5B, 5C, 5F, 5G, 5I and 5J; 6B, 6D, 6F, 6H and 6I; 7A, 7C and 7D; 8A, 8B, 8C and 8D; 9A, 9B, 9C, 9E, 9F and 9G; S2A; S5A and S5B; S8C. (XLSX)

## References

[1] Cornely OA, Sprute R, Bassetti M, Chen SC-A, Groll AH, Kurzai O, et al. Global guideline for the diagnosis and management of candidiasis: an initiative of the ECMM in cooperation with ISHAM and ASM. Lancet Infect Dis. 2025;25(5):e280–93. doi: 10.1016/S1473-3099(24)00749-7 39956121

[2] Brown GD, Denning DW, Gow NAR, Levitz SM, Netea MG, White TC. Hidden killers: human fungal infections. Sci Transl Med. 2012;4(165):165rv13. doi: 10.1126/scitranslmed.3004404 23253612

[3] Wisplinghoff H, Seifert H, Tallent SM, Bischoff T, Wenzel RP, Edmond MB. Nosocomial bloodstream infections in pediatric patients in United States hospitals: epidemiology, clinical features and susceptibilities. Pediatr Infect Dis J. 2003;22(8):686–91. doi: 10.1097/01.inf.0000078159.53132.40 12913767

[4] Dadar M, Tiwari R, Karthik K, Chakraborty S, Shahali Y, Dhama K. Candida albicans - Biology, molecular characterization, pathogenicity, and advances in diagnosis and control - An update. Microb Pathog. 2018;117:128–38. doi: 10.1016/j.micpath.2018.02.028 29454824

[5] Kullberg BJ, Arendrup MC. Invasive Candidiasis. N Engl J Med. 2015;373(15):1445–56. doi: 10.1056/NEJMra1315399 26444731

[6] Seagle EE, Jackson BR, Lockhart SR, Georgacopoulos O, Nunnally NS, Roland J, et al. The Landscape of Candidemia During the Coronavirus Disease 2019 (COVID-19) Pandemic. Clin Infect Dis. 2022;74(5):802–11. doi: 10.1093/cid/ciab562 34145450

[7] Mba IE, Nweze EI. Mechanism of Candida pathogenesis: revisiting the vital drivers. Eur J Clin Microbiol Infect Dis. 2020;39(10):1797–819. doi: 10.1007/s10096-020-03912-w 32372128

[8] Magill SS, O’Leary E, Janelle SJ, Thompson DL, Dumyati G, Nadle J, et al. Changes in Prevalence of Health Care-Associated Infections in U.S. Hospitals. N Engl J Med. 2018;379(18):1732–44. doi: 10.1056/NEJMoa1801550 30380384 PMC7978499

[9] Lass-Flörl C, Kanj SS, Govender NP, Thompson GR 3rd, Ostrosky-Zeichner L, Govrins MA. Invasive candidiasis. Nat Rev Dis Primers. 2024;10(1):20. doi: 10.1038/s41572-024-00503-3 38514673

[10] Burki T. WHO publish fungal priority pathogens list. Lancet Microbe. 2023;4(2):e74. doi: 10.1016/S2666-5247(23)00003-4 36634695

[11] Pappas PG, Lionakis MS, Arendrup MC, Ostrosky-Zeichner L, Kullberg BJ. Invasive candidiasis. Nat Rev Dis Primers. 2018;4:18026. doi: 10.1038/nrdp.2018.26 29749387

[12] Ramani K, Garg AV, Jawale CV, Conti HR, Whibley N, Jackson EK, et al. The Kallikrein-Kinin System: A Novel Mediator of IL-17-Driven Anti-Candida Immunity in the Kidney. PLoS Pathog. 2016;12(11):e1005952. doi: 10.1371/journal.ppat.1005952 27814401 PMC5096720

[13] Fan D, Coughlin LA, Neubauer MM, Kim J, Kim MS, Zhan X, et al. Activation of HIF-1α and LL-37 by commensal bacteria inhibits Candida albicans colonization. Nat Med. 2015;21(7):808–14. doi: 10.1038/nm.3871 26053625 PMC4496259

[14] Netea MG, Joosten LAB, van der Meer JWM, Kullberg B-J, van de Veerdonk FL. Immune defence against Candida fungal infections. Nat Rev Immunol. 2015;15(10):630–42. doi: 10.1038/nri3897 26388329

[15] Delsing CE, Gresnigt MS, Leentjens J, Preijers F, Frager FA, Kox M, et al. Interferon-gamma as adjunctive immunotherapy for invasive fungal infections: a case series. BMC Infect Dis. 2014;14:166. doi: 10.1186/1471-2334-14-166 24669841 PMC3987054

[16] Bär E, Whitney PG, Moor K, Reis e Sousa C, LeibundGut-Landmann S. IL-17 regulates systemic fungal immunity by controlling the functional competence of NK cells. Immunity. 2014;40(1):117–27. doi: 10.1016/j.immuni.2013.12.002 24412614

[17] Huang W, Na L, Fidel PL, Schwarzenberger P. Requirement of interleukin-17A for systemic anti-Candida albicans host defense in mice. J Infect Dis. 2004;190(3):624–31. doi: 10.1086/422329 15243941

[18] van de Veerdonk FL, Kullberg BJ, Verschueren IC, Hendriks T, van der Meer JWM, Joosten LAB, et al. Differential effects of IL-17 pathway in disseminated candidiasis and zymosan-induced multiple organ failure. Shock. 2010;34(4):407–11. doi: 10.1097/SHK.0b013e3181d67041 20160669

[19] Castellano-Gonzalez G, Clancy LE, Gottlieb D. Prospects for adoptive T-cell therapy for invasive fungal disease. Curr Opin Infect Dis. 2017;30(6):518–27. doi: 10.1097/QCO.0000000000000403 28984641

[20] Armstrong-James D, Brown GD, Netea MG, Zelante T, Gresnigt MS, van de Veerdonk FL, et al. Immunotherapeutic approaches to treatment of fungal diseases. Lancet Infect Dis. 2017;17(12):e393–402. doi: 10.1016/S1473-3099(17)30442-5 28774700

[21] Labrijn AF, Janmaat ML, Reichert JM, Parren PWHI. Bispecific antibodies: a mechanistic review of the pipeline. Nat Rev Drug Discov. 2019;18(8):585–608. doi: 10.1038/s41573-019-0028-1 31175342

[22] Davenport AJ, Jenkins MR. Programming a serial killer: CAR T cells form non-classical immune synapses. Oncoscience. 2018;5(3–4):69–70. doi: 10.18632/oncoscience.406 29854873 PMC5978443

[23] Offner S, Hofmeister R, Romaniuk A, Kufer P, Baeuerle PA. Induction of regular cytolytic T cell synapses by bispecific single-chain antibody constructs on MHC class I-negative tumor cells. Mol Immunol. 2006;43(6):763–71. doi: 10.1016/j.molimm.2005.03.007 16360021

[24] Hoffmann P, Hofmeister R, Brischwein K, Brandl C, Crommer S, Bargou R, et al. Serial killing of tumor cells by cytotoxic T cells redirected with a CD19-/CD3-bispecific single-chain antibody construct. Int J Cancer. 2005;115(1):98–104. doi: 10.1002/ijc.20908 15688411

[25] Quitt O, Luo S, Meyer M, Xie Z, Golsaz-Shirazi F, Loffredo-Verde E, et al. T-cell engager antibodies enable T cells to control HBV infection and to target HBsAg-positive hepatoma in mice. J Hepatol. 2021;75(5):1058–71. doi: 10.1016/j.jhep.2021.06.022 34171437

[26] Sloan DD, Lam C-YK, Irrinki A, Liu L, Tsai A, Pace CS, et al. Targeting HIV Reservoir in Infected CD4 T Cells by Dual-Affinity Re-targeting Molecules (DARTs) that Bind HIV Envelope and Recruit Cytotoxic T Cells. PLoS Pathog. 2015;11(11):e1005233. doi: 10.1371/journal.ppat.1005233 26539983 PMC4634948

[27] Kruse RL, Shum T, Legras X, Barzi M, Pankowicz FP, Gottschalk S, et al. In Situ Liver Expression of HBsAg/CD3-Bispecific Antibodies for HBV Immunotherapy. Mol Ther Methods Clin Dev. 2017;7:32–41. doi: 10.1016/j.omtm.2017.08.006 29018834 PMC5626922

[28] Gantner BN, Simmons RM, Underhill DM. Dectin-1 mediates macrophage recognition of Candida albicans yeast but not filaments. EMBO J. 2005;24(6):1277–86. doi: 10.1038/sj.emboj.7600594 15729357 PMC556398

[29] Netea MG, Brown GD, Kullberg BJ, Gow NAR. An integrated model of the recognition of Candida albicans by the innate immune system. Nat Rev Microbiol. 2008;6(1):67–78. doi: 10.1038/nrmicro1815 18079743

[30] Ariizumi K, Shen GL, Shikano S, Xu S, Ritter R 3rd, Kumamoto T, et al. Identification of a novel, dendritic cell-associated molecule, dectin-1, by subtractive cDNA cloning. J Biol Chem. 2000;275(26):20157–67. doi: 10.1074/jbc.M909512199 10779524

[31] Taylor PR, Brown GD, Reid DM, Willment JA, Martinez-Pomares L, Gordon S, et al. The beta-glucan receptor, dectin-1, is predominantly expressed on the surface of cells of the monocyte/macrophage and neutrophil lineages. J Immunol. 2002;169(7):3876–82. doi: 10.4049/jimmunol.169.7.3876 12244185

[32] Yokota K, Takashima A, Bergstresser PR, Ariizumi K. Identification of a human homologue of the dendritic cell-associated C-type lectin-1, dectin-1. Gene. 2001;272(1–2):51–60. doi: 10.1016/s0378-1119(01)00528-5 11470510

[33] Wang Z, Zhu J, Lu H. Antibody glycosylation: impact on antibody drug characteristics and quality control. Appl Microbiol Biotechnol. 2020;104(5):1905–14. doi: 10.1007/s00253-020-10368-7 31940081

[34] Willment JA, Gordon S, Brown GD. Characterization of the human beta -glucan receptor and its alternatively spliced isoforms. J Biol Chem. 2001;276(47):43818–23. doi: 10.1074/jbc.M107715200 11567029

[35] Zhang SQ, Zou Z, Shen H, Shen SS, Miao Q, Huang X, et al. Mnn10 Maintains Pathogenicity in Candida albicans by Extending α-1,6-Mannose Backbone to Evade Host Dectin-1 Mediated Antifungal Immunity. PLoS Pathog. 2016;12(5):e1005617. doi: 10.1371/journal.ppat.1005617 27144456 PMC4856274

[36] Hezareh M, Hessell AJ, Jensen RC, van de Winkel JG, Parren PW. Effector function activities of a panel of mutants of a broadly neutralizing antibody against human immunodeficiency virus type 1. J Virol. 2001;75(24):12161–8. doi: 10.1128/JVI.75.24.12161-12168.2001 11711607 PMC116112

[37] Borghi M, Renga G, Puccetti M, Oikonomou V, Palmieri M, Galosi C, et al. Antifungal Th Immunity: Growing up in Family. Front Immunol. 2014;5:506. doi: 10.3389/fimmu.2014.00506 25360137 PMC4197763

[38] Iliev ID, Leonardi I. Fungal dysbiosis: immunity and interactions at mucosal barriers. Nat Rev Immunol. 2017;17(10):635–46. doi: 10.1038/nri.2017.55 28604735 PMC5724762

[39] Weaver CT, Hatton RD, Mangan PR, Harrington LE. IL-17 family cytokines and the expanding diversity of effector T cell lineages. Annu Rev Immunol. 2007;25:821–52. doi: 10.1146/annurev.immunol.25.022106.141557 17201677

[40] Norelli M, Camisa B, Barbiera G, Falcone L, Purevdorj A, Genua M, et al. Monocyte-derived IL-1 and IL-6 are differentially required for cytokine-release syndrome and neurotoxicity due to CAR T cells. Nat Med. 2018;24(6):739–48. doi: 10.1038/s41591-018-0036-4 29808007

[41] McDermott DS, Varga SM. Quantifying antigen-specific CD4 T cells during a viral infection: CD4 T cell responses are larger than we think. J Immunol. 2011;187(11):5568–76. doi: 10.4049/jimmunol.1102104 22043009 PMC3221938

[42] Christiaansen AF, Dixit UG, Coler RN, Marie Beckmann A, Reed SG, Winokur PL, et al. CD11a and CD49d enhance the detection of antigen-specific T cells following human vaccination. Vaccine. 2017;35(33):4255–61. doi: 10.1016/j.vaccine.2017.06.013 28662951 PMC5551405

[43] Butler NS, Moebius J, Pewe LL, Traore B, Doumbo OK, Tygrett LT, et al. Therapeutic blockade of PD-L1 and LAG-3 rapidly clears established blood-stage Plasmodium infection. Nat Immunol. 2011;13(2):188–95. doi: 10.1038/ni.2180 22157630 PMC3262959

[44] Pessano S, Oettgen H, Bhan AK, Terhorst C. The T3/T cell receptor complex: antigenic distinction between the two 20-kd T3 (T3-delta and T3-epsilon) subunits. EMBO J. 1985;4(2):337–44. doi: 10.1002/j.1460-2075.1985.tb03634.x 2410254 PMC554191

[45] Fenis A, Demaria O, Gauthier L, Vivier E, Narni-Mancinelli E. New immune cell engagers for cancer immunotherapy. Nat Rev Immunol. 2024;24(7):471–86. doi: 10.1038/s41577-023-00982-7 38273127

[46] Bortoletto N, Scotet E, Myamoto Y, D’Oro U, Lanzavecchia A. Optimizing anti-CD3 affinity for effective T cell targeting against tumor cells. Eur J Immunol. 2002;32(11):3102–7. doi: 10.1002/1521-4141(200211)32:11<3102::AID-IMMU3102>3.0.CO;2-C 12385030

[47] List T, Neri D. Biodistribution studies with tumor-targeting bispecific antibodies reveal selective accumulation at the tumor site. MAbs. 2012;4(6):775–83. doi: 10.4161/mabs.22271 23032949 PMC3502244

[48] Mandikian D, Takahashi N, Lo AA, Li J, Eastham-Anderson J, Slaga D, et al. Relative Target Affinities of T-Cell-Dependent Bispecific Antibodies Determine Biodistribution in a Solid Tumor Mouse Model. Mol Cancer Ther. 2018;17(4):776–85. doi: 10.1158/1535-7163.MCT-17-0657 29339550

[49] Ravikumar S, Win MS, Chai LYA. Optimizing Outcomes in Immunocompromised Hosts: Understanding the Role of Immunotherapy in Invasive Fungal Diseases. Front Microbiol. 2015;6:1322. doi: 10.3389/fmicb.2015.01322 26635780 PMC4660869

[50] Goebeler M-E, Bargou RC. T cell-engaging therapies - BiTEs and beyond. Nat Rev Clin Oncol. 2020;17(7):418–34. doi: 10.1038/s41571-020-0347-5 32242094

[51] van de Donk NWCJ, Zweegman S. T-cell-engaging bispecific antibodies in cancer. Lancet. 2023;402(10396):142–58. doi: 10.1016/S0140-6736(23)00521-4 37271153

[52] Takano T, Motozono C, Imai T, Sonoda K-H, Nakanishi Y, Yamasaki S. Dectin-1 intracellular domain determines species-specific ligand spectrum by modulating receptor sensitivity. J Biol Chem. 2017;292(41):16933–41. doi: 10.1074/jbc.M117.800847 28848046 PMC5641891

[53] Illiano A, Pinto G, Melchiorre C, Carpentieri A, Faraco V, Amoresano A. Protein Glycosylation Investigated by Mass Spectrometry: An Overview. Cells. 2020;9(9):1986. doi: 10.3390/cells9091986 32872358 PMC7564411

[54] Romani L. Immunity to fungal infections. Nat Rev Immunol. 2011;11(4):275–88. doi: 10.1038/nri2939 21394104

[55] Wüthrich M, Deepe GS Jr, Klein B. Adaptive immunity to fungi. Annu Rev Immunol. 2012;30:115–48. doi: 10.1146/annurev-immunol-020711-074958 22224780 PMC3584681

[56] Ouyang W, O’Garra A. IL-10 Family Cytokines IL-10 and IL-22: from Basic Science to Clinical Translation. Immunity. 2019;50(4):871–91. doi: 10.1016/j.immuni.2019.03.020 30995504

[57] Drummond RA, Wallace C, Reid DM, Way SS, Kaplan DH, Brown GD. Cutting edge: Failure of antigen-specific CD4+ T cell recruitment to the kidney during systemic candidiasis. J Immunol. 2014;193(11):5381–5. doi: 10.4049/jimmunol.1401675 25344471 PMC4238746

[58] Drummond RA, Desai JV, Ricotta EE, Swamydas M, Deming C, Conlan S, et al. Long-term antibiotic exposure promotes mortality after systemic fungal infection by driving lymphocyte dysfunction and systemic escape of commensal bacteria. Cell Host Microbe. 2022;30(7):1020-1033.e6. doi: 10.1016/j.chom.2022.04.013 35568028 PMC9283303

[59] Li M, Li C, Wu X, Chen T, Ren L, Xu B, et al. Microbiota-driven interleukin-17 production provides immune protection against invasive candidiasis. Crit Care. 2020;24(1):268. doi: 10.1186/s13054-020-02977-5 32460890 PMC7251893

[60] Koh AY, Köhler JR, Coggshall KT, Van Rooijen N, Pier GB. Mucosal damage and neutropenia are required for Candida albicans dissemination. PLoS Pathog. 2008;4(2):e35. doi: 10.1371/journal.ppat.0040035 18282097 PMC2242836

[61] Odds FC, Davidson AD, Jacobsen MD, Tavanti A, Whyte JA, Kibbler CC, et al. Candida albicans strain maintenance, replacement, and microvariation demonstrated by multilocus sequence typing. J Clin Microbiol. 2006;44(10):3647–58. doi: 10.1128/JCM.00934-06 17021093 PMC1594753

[62] Zhai B, Ola M, Rolling T, Tosini NL, Joshowitz S, Littmann ER, et al. High-resolution mycobiota analysis reveals dynamic intestinal translocation preceding invasive candidiasis. Nat Med. 2020;26(1):59–64. doi: 10.1038/s41591-019-0709-7 31907459 PMC7005909

[63] Iliev ID, Funari VA, Taylor KD, Nguyen Q, Reyes CN, Strom SP, et al. Interactions between commensal fungi and the C-type lectin receptor Dectin-1 influence colitis. Science. 2012;336(6086):1314–7. doi: 10.1126/science.1221789 22674328 PMC3432565

[64] Rapaka RR, Goetzman ES, Zheng M, Vockley J, McKinley L, Kolls JK, et al. Enhanced defense against Pneumocystis carinii mediated by a novel dectin-1 receptor Fc fusion protein. J Immunol. 2007;178(6):3702–12. doi: 10.4049/jimmunol.178.6.3702 17339468

[65] Kumaresan PR, Manuri PR, Albert ND, Maiti S, Singh H, Mi T, et al. Bioengineering T cells to target carbohydrate to treat opportunistic fungal infection. Proc Natl Acad Sci U S A. 2014;111(29):10660–5. doi: 10.1073/pnas.1312789111 25002471 PMC4115509

[66] Shimabukuro-Vornhagen A, Gödel P, Subklewe M, Stemmler HJ, Schlößer HA, Schlaak M, et al. Cytokine release syndrome. J Immunother Cancer. 2018;6(1):56. doi: 10.1186/s40425-018-0343-9 29907163 PMC6003181

[67] Leong SR, Sukumaran S, Hristopoulos M, Totpal K, Stainton S, Lu E, et al. An anti-CD3/anti-CLL-1 bispecific antibody for the treatment of acute myeloid leukemia. Blood. 2017;129(5):609–18. doi: 10.1182/blood-2016-08-735365 27908880 PMC5290988

[68] Wu Z, Cheung NV. T cell engaging bispecific antibody (T-BsAb): From technology to therapeutics. Pharmacol Ther. 2018;182:161–75. doi: 10.1016/j.pharmthera.2017.08.005 28834699 PMC5785550

[69] Santich BH, Park JA, Tran H, Guo H-F, Huse M, Cheung N-KV. Interdomain spacing and spatial configuration drive the potency of IgG-[L]-scFv T cell bispecific antibodies. Sci Transl Med. 2020;12(534):eaax1315. doi: 10.1126/scitranslmed.aax1315 32161106 PMC7437947

