# Supplementary figures and images for: Chimeric PRR T-cell-engager targeting cell surface β-1,3-glucan for invasive candidiasis
Source: PLoS Pathog. 2025 Sep 15;21(9):e1013508. doi: 10.1371/journal.ppat.1013508 (PMC12478890; doi:10.1371/journal.ppat.1013508)

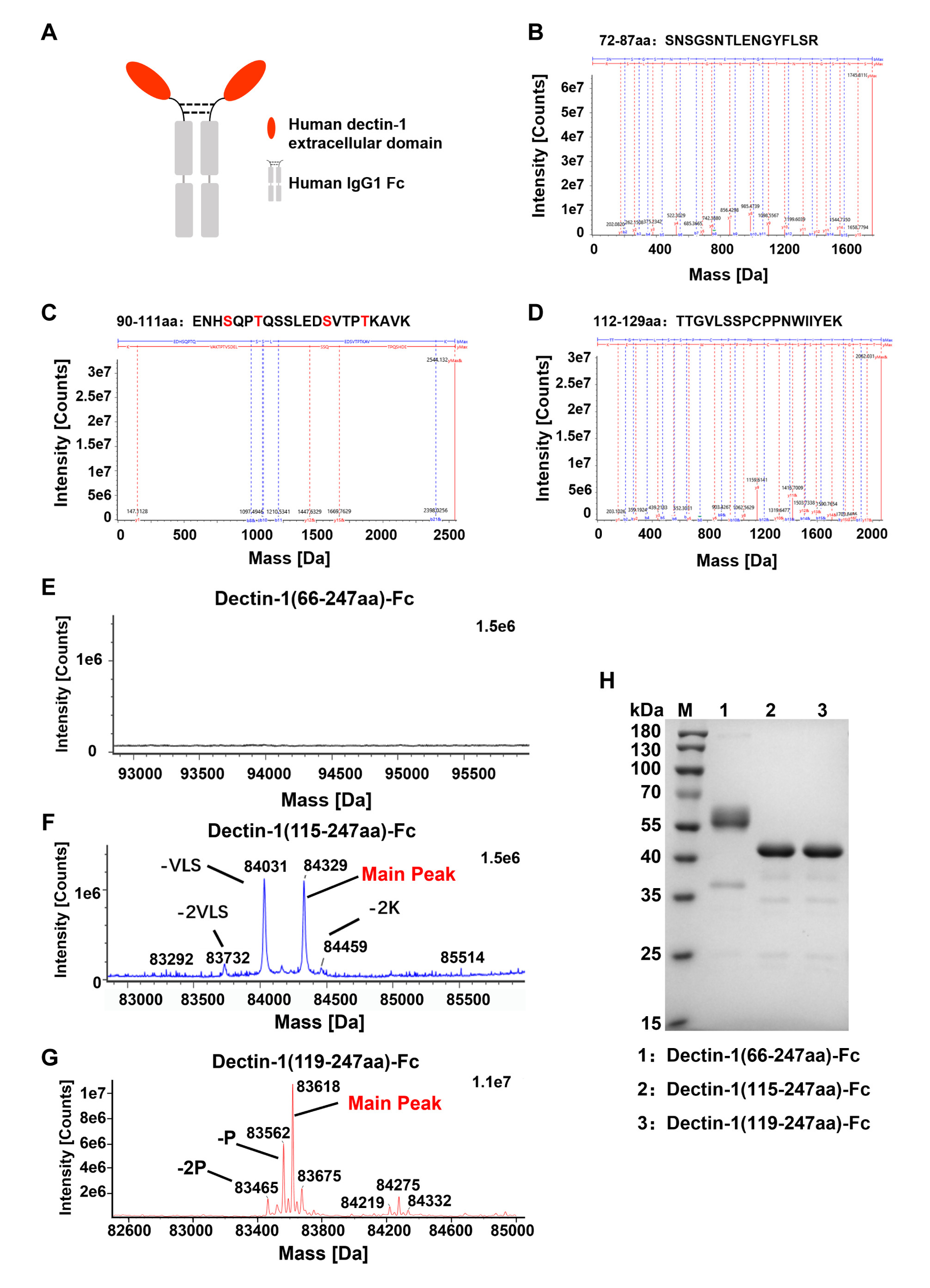

Supplement: S1 Fig — (A) Schematic depiction of dectin-1-Fc fusion protein. (B-D) Mass spectrometry analysis of dectin-1(66–247aa)-Fc peptide fragments spanning 72–87aa (B), 90–111aa (C), and 112–129aa (D). (E-G) Molecular weights of dectin-1(66–247aa)-Fc (E), dectin-1(115–247aa)-Fc (F), and dectin-1(119–247aa)-Fc (G) were analyzed by mass spectrometry. (H) Non-reducing SDS-PAGE of dectin-1-Fc fusion proteins. Data are representative of three independent experiments. SDS-PAGE, sodium dodecyl sulfate polyacrylamide gel electrophoresis. (TIF) [file ppat.1013508.s001.tif]

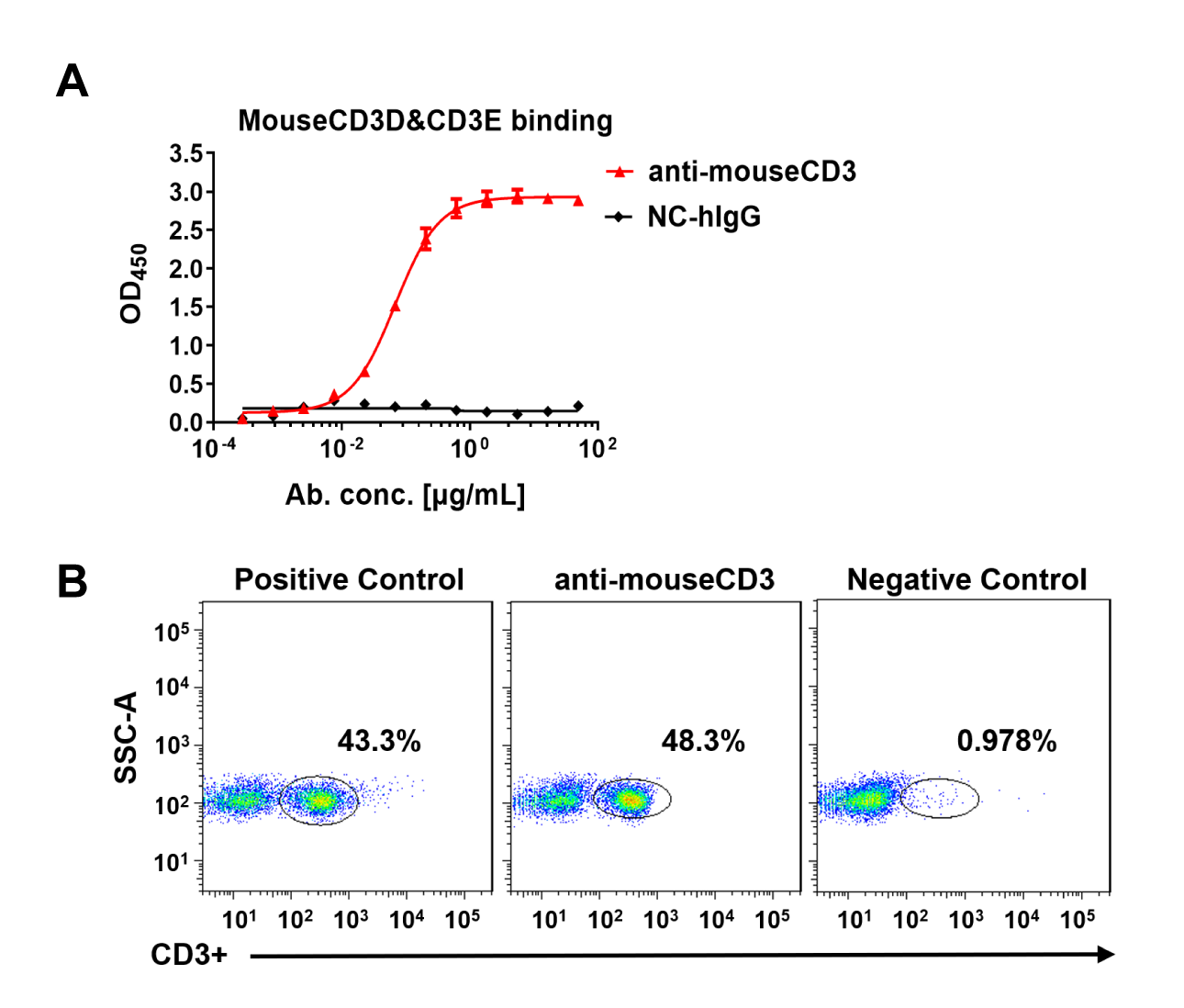

Supplement: S2 Fig — (A) ELISA assay for anti-mouseCD3 binding to mouseCD3D&CD3E protein. Data are means ± SD (n = 2) and are representative of three independent experiments. (B) Representative images analysed by flow cytometry for detection of anti-mouseCD3 to murine splenocytes. Data are representative of three independent experiments; Commercialized APC/Cyanine7 anti-mouseCD3 antibody served as positive control; isotype unrelated antibody served as a negative control; ELISA, enzyme-linked immunosorbent assay. (TIF) [file ppat.1013508.s002.tif]

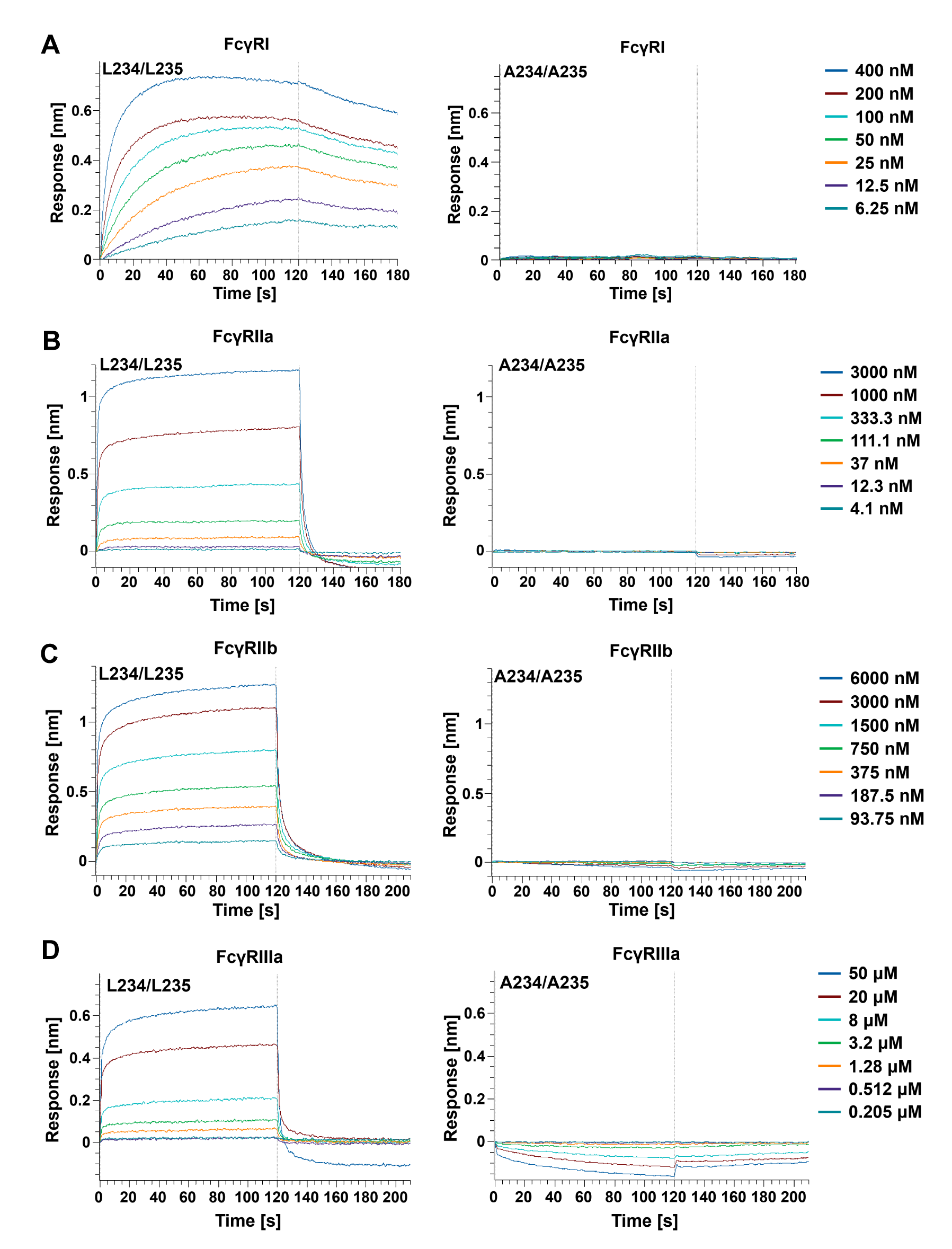

Supplement: S3 Fig — BLI analysis for the binding between mXJ104 and FcγRI (A), FcγRIIa (B), FcγRIIb (C) and FcγRIIIa (D). L234/L235 (left), mXJ104 without the double mutant; A234/A235 (right), mXJ104 with the double mutant. Data are representative of three independent experiments. BLI, Bio-Layer Interferometry. (TIF) [file ppat.1013508.s003.tif]

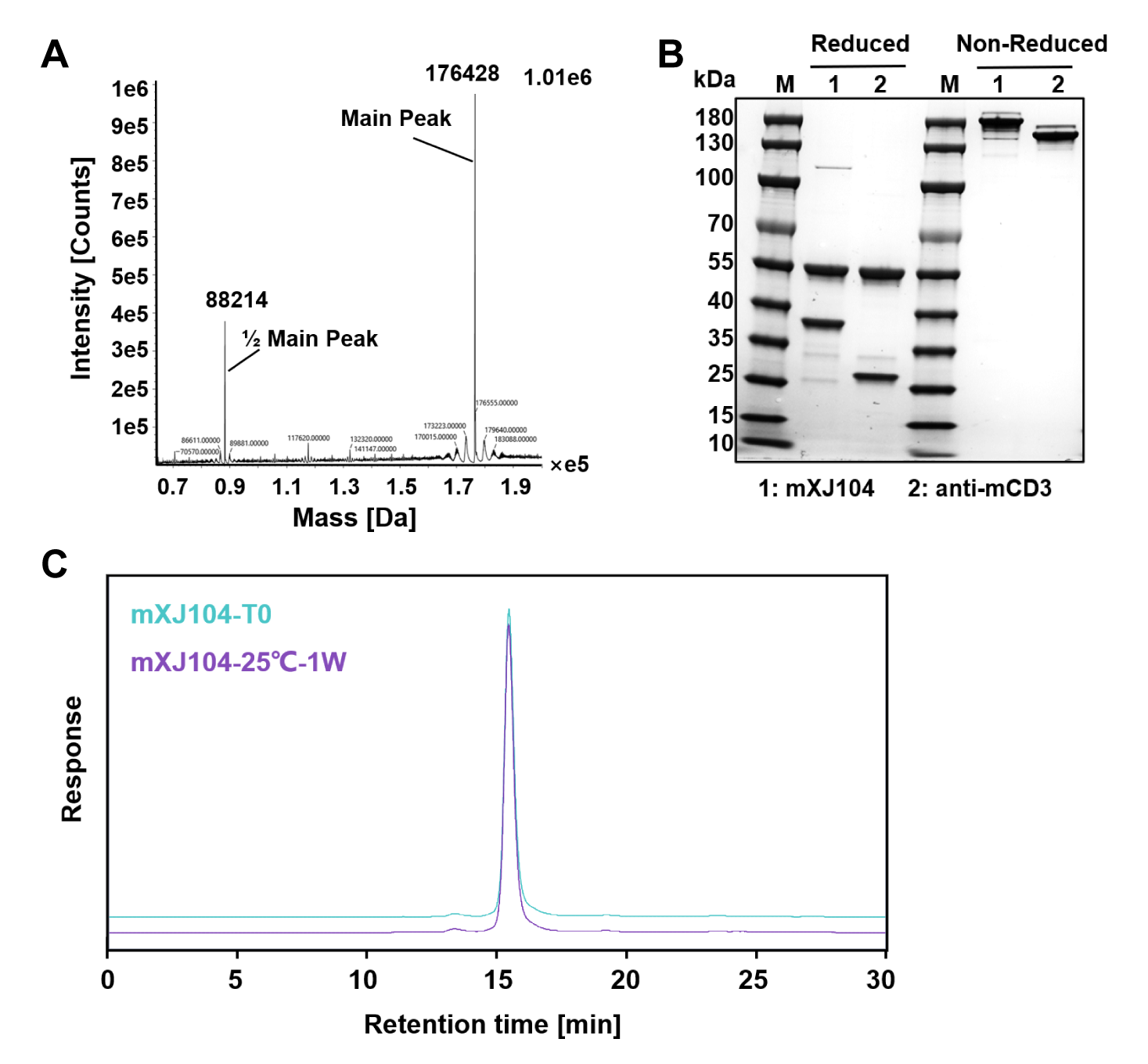

Supplement: S4 Fig — (A-B) Molecular weight of anti-mCD3-dectin-1(119–247aa) (mXJ104), as confirmed by mass spectrometer (A) and SDS-PAGE (B). (C) Thermal stability analysis of mXJ104 by SEC. Data are representative of three independent experiments (A, B, C). SDS-PAGE, sodium dodecyl sulfate polyacrylamide gel electrophoresis; SEC, size exclusion chromatography. (TIF) [file ppat.1013508.s004.tif]

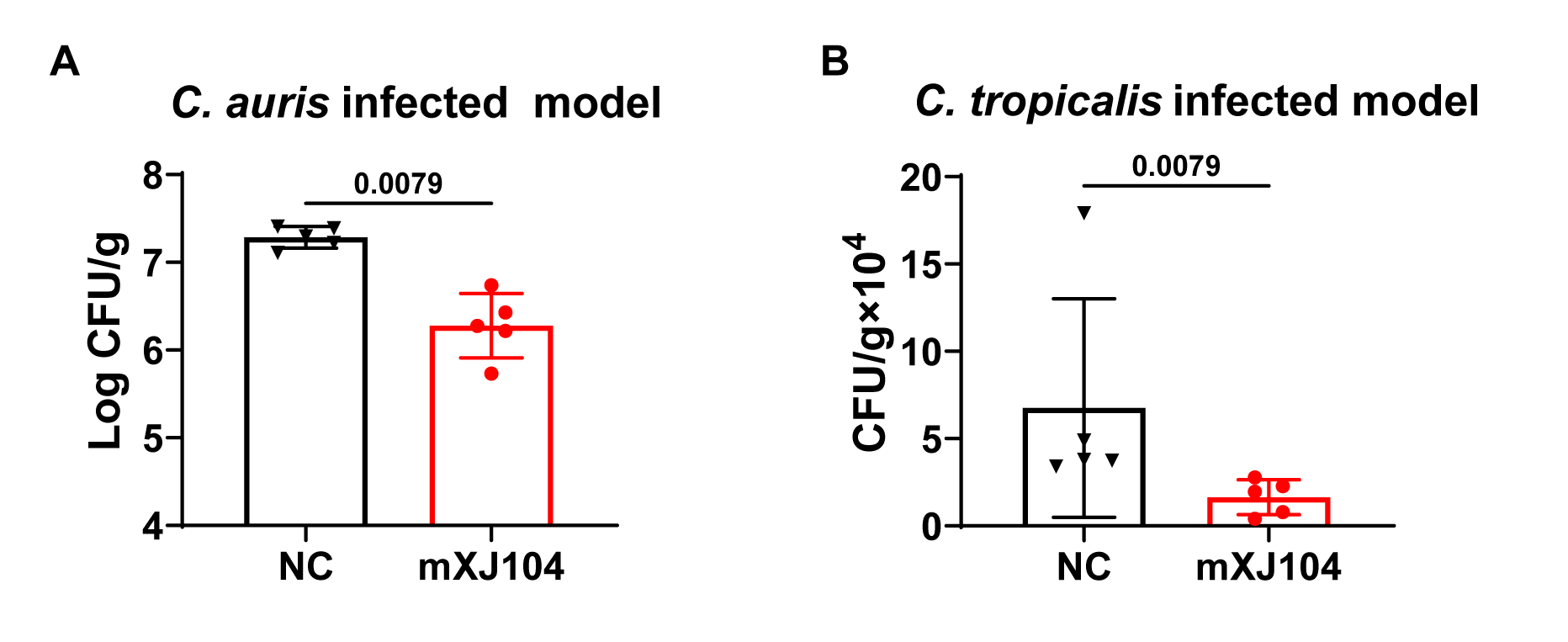

Supplement: S5 Fig — C57BL/6 mice were intravenously infected with C. auris 1212 (3 × 10^7^ CFU) and C. tropicalis ATCC750 (1 × 10^6^ CFU), and treated with mXJ104 or negative control antibody (1 mg/kg). Quantification of the fungal burden in kidneys at 48 hours post-treatment. The kidney fungal burden of mice infected with C. auris 1212 (A) or C. tropicalis ATCC750 (B), n = 5. Data are representative of three independent experiments (A, B); **, P < 0.01, Kolmogorov-Smirnov nonparametric test (A, B). (TIF) [file ppat.1013508.s005.tif]

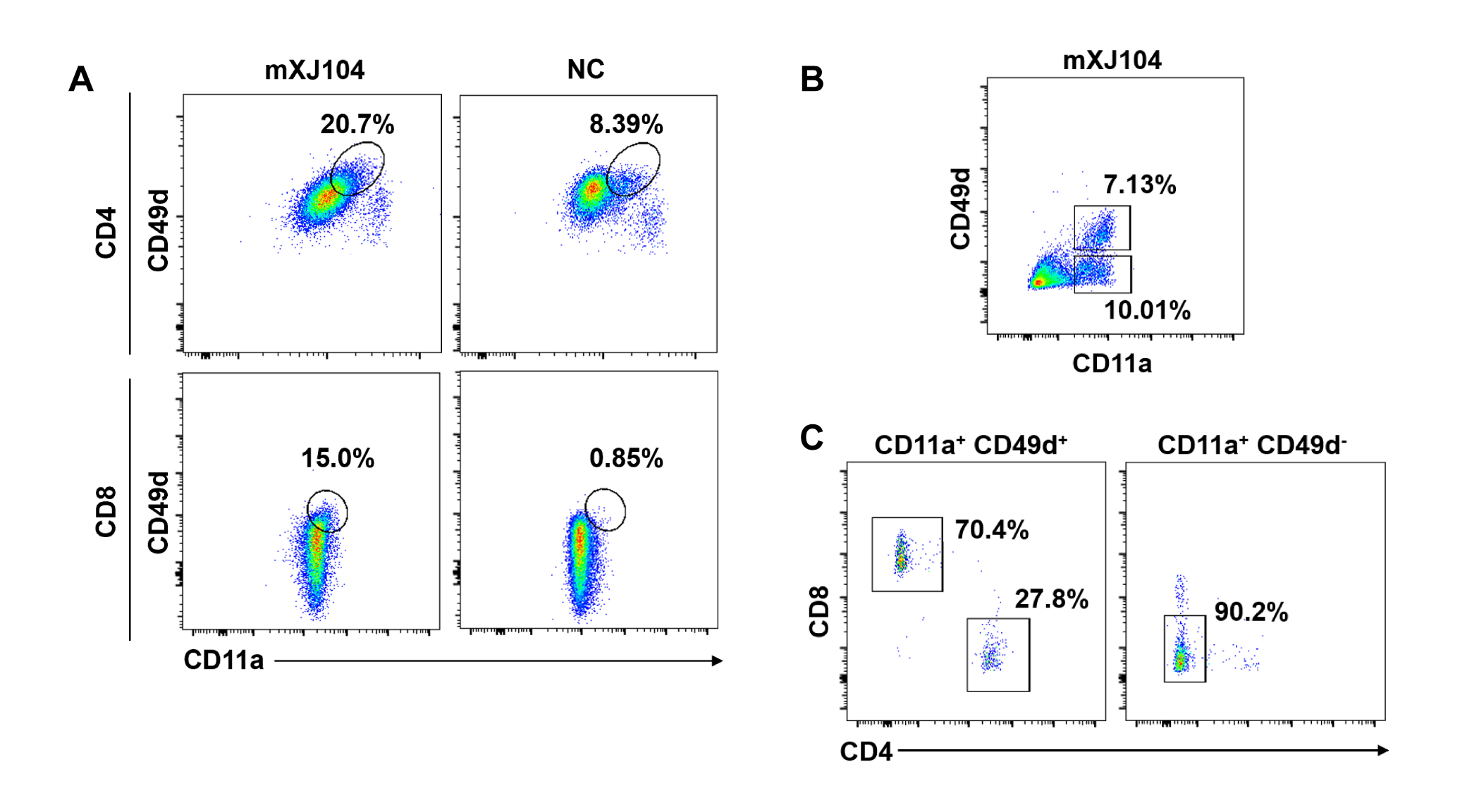

Supplement: S6 Fig — C57BL/6 mice were intravenously infected with C. albicans SC5314 (1 × 10^5^ CFU, defined as day -5) and treated with mXJ104 or NC on day 0, respectively. (A) Representive CD11a and CD49d expression on CD4+ (Top panel) and CD8+ (bottom panel) CD3+ T cells in spleen from infected mice treated with mXJ104 (left panel) or NC (right panel). (B) Frequency of CD11a+ CD49d+ cells in kidneys from infected mice treated with mXJ104. (C) Frequency of CD8+ or CD4+ CD3+ T cells in CD11a+ CD49d+ (left panel) or CD11a+ CD49d- (right panel) cells. Spleens and kidneys were harvested at 48 hours post-treatment, and data are representative image of five mice (A, B, C). (TIF) [file ppat.1013508.s006.tif]

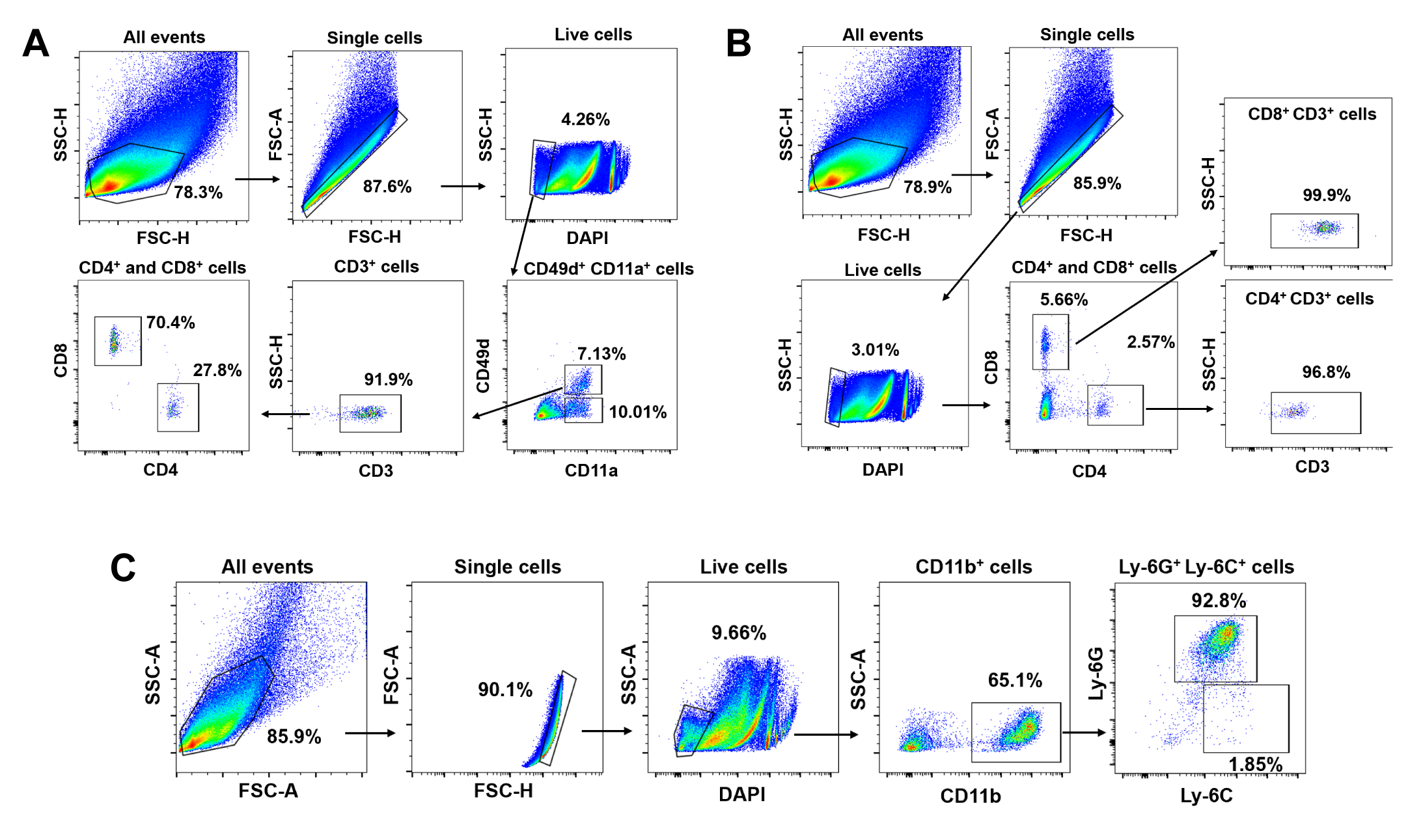

Supplement: S7 Fig — Single cells were first gated from all events obtained from kidneys of mXJ104-treated infected mice, followed by the selection of live cells from the single-cell gate. (A) CD49d ⁺ CD11a⁺ cells were gated from the live cells. This population was confirmed to be CD3⁺ and composed of CD4⁺ and CD8 ⁺ T cells. (B) From live cells, CD4+ and CD8+ cells were gated and confirmed to be CD3+ T cells. (C) CD11b⁺ cells were gated from the live cells. This population was confirmed to consist predominantly of Ly-6G ⁺ Ly-6C⁺ cells. (TIF) [file ppat.1013508.s007.tif]

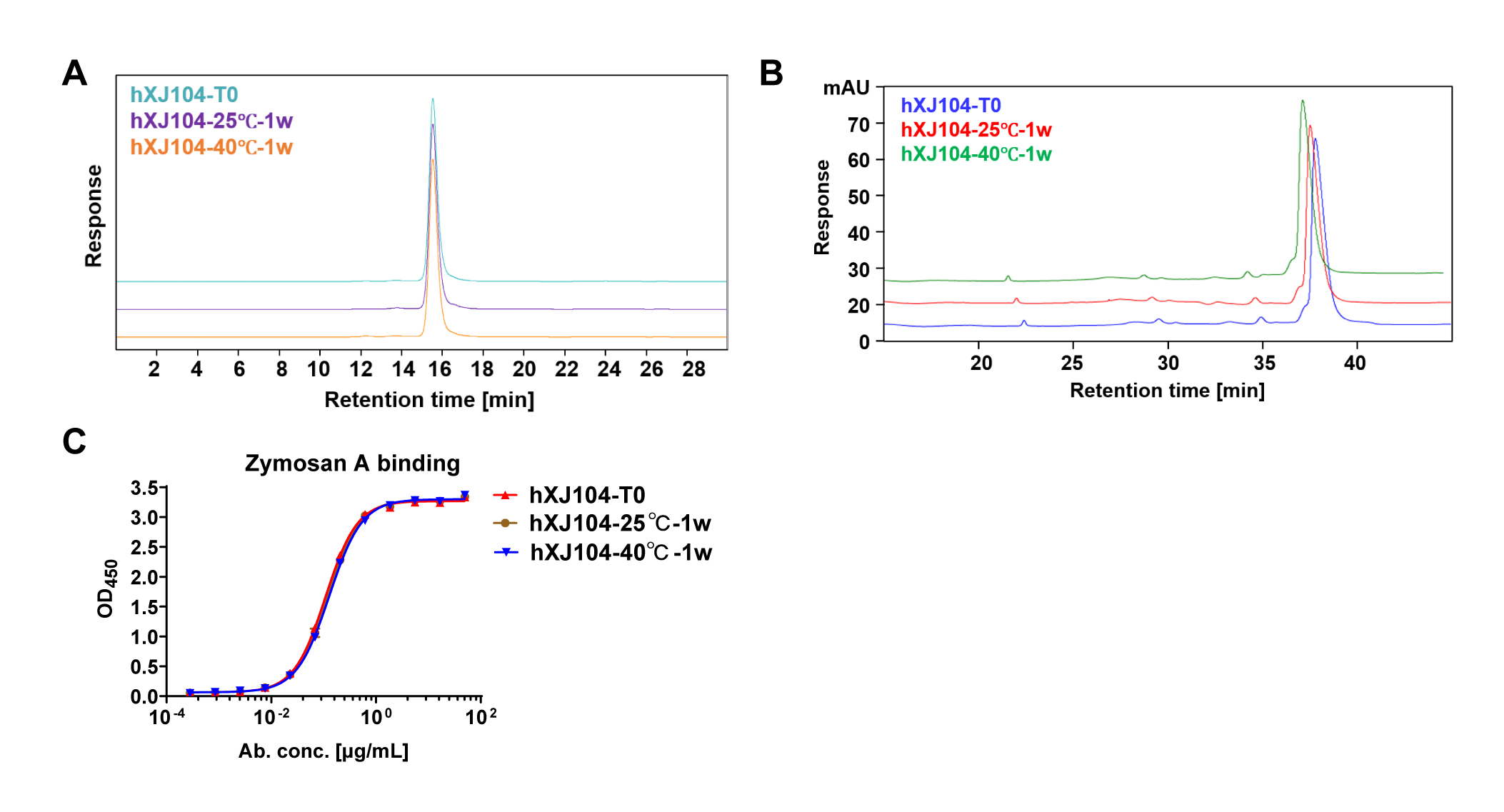

Supplement: S8 Fig — (A-B) Purity assessment of mXJ104 stability samples by SEC (A) and NR-CE (B). Data are representative of three independent experiments. (C) The zymosan A-binding activity of mXJ104 stability samples was evaluated by ELISA, Data are means ± SD (n = 2) and are representative of three independent experiments. SEC, size-exclusion chromatography; NR-CE, non-reduced capillary electrophoresis; ELISA, enzyme-linked immunosorbent assay. (TIF) [file ppat.1013508.s008.tif]

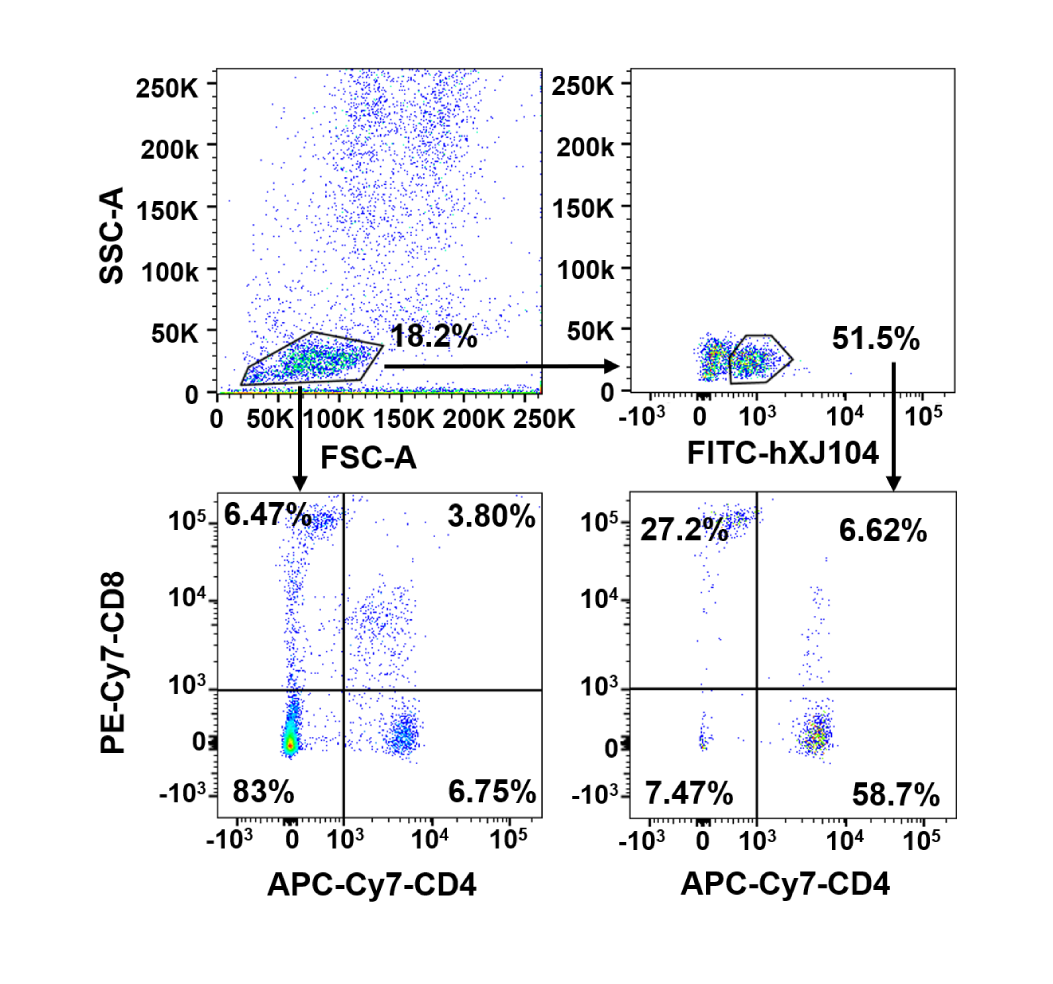

Supplement: S9 Fig — Schematic of flow cytometry analysis from representative sample (n = 10). Firstly, live cells were gated, and then cells reactive to hXJ104 were gated. Subsequently, CD4+ and CD8+ cell subsets were also gated in hXJ104 reactive cells and all live cells. (TIF) [file ppat.1013508.s009.tif]
